# Arginyltransferase (Ate1) regulates the RGS7 protein level and the sensitivity of light-evoked ON-bipolar responses

**DOI:** 10.1038/s41598-021-88628-3

**Published:** 2021-04-30

**Authors:** Marie E. Fina, Junling Wang, Sergei S. Nikonov, Stephanie Sterling, Noga Vardi, Anna Kashina, Dawei W. Dong

**Affiliations:** 1 Department of Biomedical Sciences, School of Veterinary Medicines, University of Pennsylvania, Philadelphia, PA 19104 USA; 2 Department of Neuroscience, Perelman School of Medicine, University of Pennsylvania, Philadelphia, PA 19104 USA; 3 Institute for Biomedical Informatics, Perelman School of Medicine, University of Pennsylvania, Philadelphia, PA 19104 USA

**Keywords:** Cell biology, Neuroscience

## Abstract

Regulator of G-protein signaling 7 (RGS7) is predominately present in the nervous system and is essential for neuronal signaling involving G-proteins. Prior studies in cultured cells showed that RGS7 is regulated via proteasomal degradation, however no protein is known to facilitate proteasomal degradation of RGS7 and it has not been shown whether this regulation affects G-protein signaling in neurons. Here we used a knockout mouse model with conditional deletion of arginyltransferase (Ate1) in the nervous system and found that in retinal ON bipolar cells, where RGS7 modulates a G-protein to signal light increments, deletion of Ate1 raised the level of RGS7. Electroretinographs revealed that lack of Ate1 leads to increased light-evoked response sensitivities of ON-bipolar cells, as well as their downstream neurons. In cultured mouse embryonic fibroblasts (MEF), RGS7 was rapidly degraded via proteasome pathway and this degradation was abolished in Ate1 knockout MEF. Our results indicate that Ate1 regulates RGS7 protein level by facilitating proteasomal degradation of RGS7 and thus affects G-protein signaling in neurons.

## Introduction

Arginyltransferase 1 (Ate1) was initially characterized as a central component of post-translational modifications of protein N-termini for ubiquitin-proteasomal degradation^1,2^. Through this N-end rule pathway^3^, Ate1 mediates the degradation of three regulator of G-protein signaling (RGS) R4 family members, RGS4, RGS5, and RGS16, in an oxidation-dependent mechanism that targets the N-terminal Cys residue in these proteins, which is exposed after physiological removal of the initiator Met^4,5^. This pathway has been implicated in heart development, which is impaired in mice upon Ate1 deletion^6^. But members of other RGS families have never been shown to be regulated by Ate1. Many studies revealed expended roles of Ate1 in a large number of key physiological processes (see, e.g.,^7–13^), including nerve regeneration^14^, neuronal stress signaling^15^, neurite outgrowth^16^, and neurodegeneration^17^. However, the roles of Ate1 in the nervous system remain poorly understood^18^, and in particular no study showed any synaptic modification or neuronal activity changes related to Ate1.

RGS proteins accelerate the termination of G-protein signaling pathways by facilitating hydrolysis of the G$\alpha$-bound GTP, thus promoting reassociation of G$\alpha$ with G$\beta \gamma$ subunits^19–24^. The effects of RGS are opposite to those of the ligand activated G-protein coupled receptors (GPCRs), which facilitate the formation of G$\alpha$-GTP and hence promote the dissociation of G$\alpha$ from G$\beta \gamma$, both of which can subsequently modulate downstream effectors. Specific RGS members carry out this signaling mechanism in different cell types. Collectively, RGS proteins are involved in virtually every known physiological process and play major roles in neuronal signal transduction^25^. Consequently they have been implicated in many neurological diseases including retinal dysfunction, amyotrophic lateral sclerosis, Parkinson’s and Alzheimer’s diseases^26^.

The most abundant and ubiquitous RGS protein in the central nervous system is RGS7, which is highly expressed in the cerebral cortex, basal ganglia, amygdala, hippocampus, hypothalamus, thalamus, brainstem, and cerebellum^27^ as well as in the retina^28^. In the retina, RGS7 accelerates the light-on responses of ON-bipolar cells at the first visual synapse^29–32^. In the retina and other brain regions, RGS7 exists as an obligatory heterodimer with G-protein subunit beta 5 (G$\beta _5$)^33–36^. In mouse retina and striatum, deleting G$\beta _5$ results in a total loss of RGS7 protein without any effect on Rgs7 mRNA levels^37^. Several studies, carried out by transfecting Rgs7 construct into cultured cells, showed that RGS7 protein is degraded rapidly by proteasomal pathways, and is stabilized by co-transfecting polycyctin 1 (Pkd1)^38^, tumor necrosis factor (Tnf)^39^, and G-protein subunit beta 5 (Gnb5)^34^. In addition, RGS7 is stabilized by other proteins involved in subcellular targeting of RGS7, including G-protein subunit alpha o (G$\alpha _{o}$)^40^, RGS7 binding protein (R7BP)^41,42^, and GPCR 158 (GPR158) or GPCR 179 (GPR179)^43^. But no protein is known to facilitate the proteasome degradation of RGS7, and no study has been done in neurons to show the regulating effect of RGS7 degradation on G-protein signaling.

Here we used the retina as a model system to investigate the role of Ate1 in neuronal responses and tested the underlying molecular mechanisms of Ate1-dependent regulation of neuronal function. Using a conditional knockout mouse model, we found that Ate1 deletion in the retina leads to increased sensitivity of light responses in the ON-bipolar cells and their downstream neurons. Analysis of the proteins involved in the ON-bipolar signaling revealed a prominent increase in the levels of RGS7 and its obligatory binding partner G$\beta _5$. Further tests showed that the increase in RGS7 is responsible for the increase of RGS7-G$\beta _5$ dimers in the knockout neurons and that RGS7 proteasomal degradation is abolished in Ate1 knockout cells. We propose that Ate1 regulates RGS7 levels by directly or indirectly facilitating proteasomal degradation of RGS7 in vivo and thus regulates RGS7-dependent G-protein signaling in the nervous system.

## Results

### Deleting Ate1 in the brain and the retina

**Figure 1:**
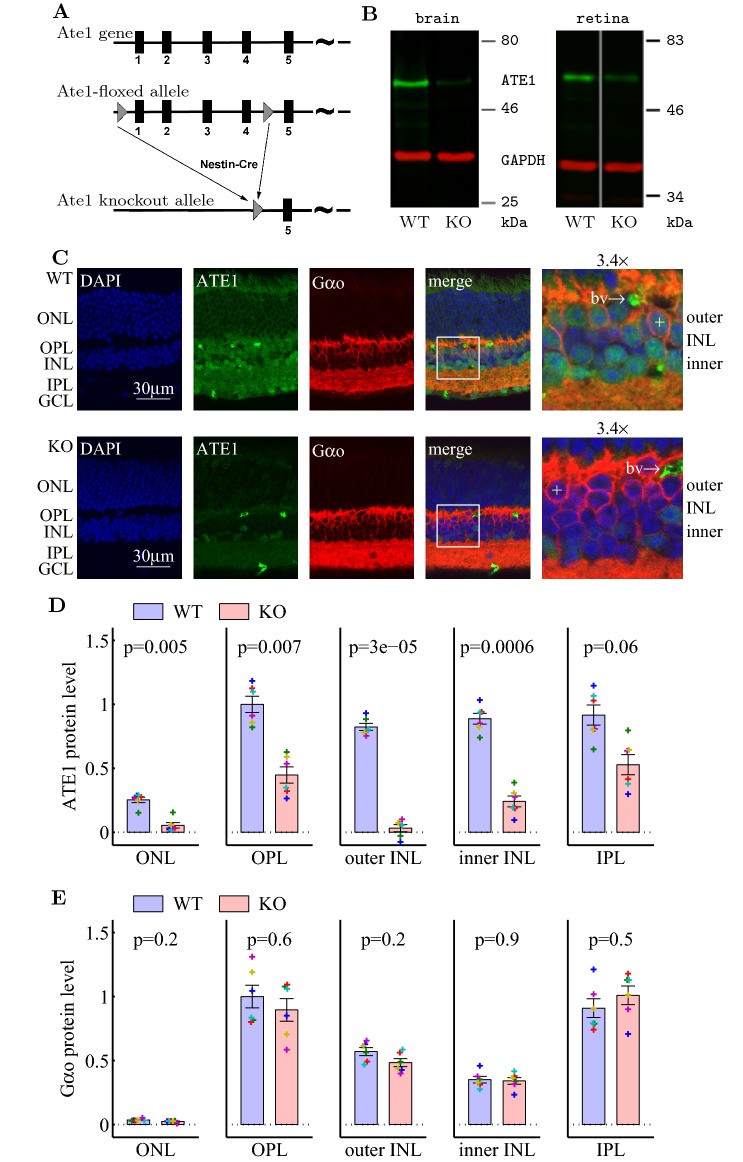
Deleting Ate1 in the brain and the retina. (**A**) Strategy for generating Ate1 conditional knockout (KO) mice. (**B**) Western blots using ATE1 antibodies confirm near-absence of ATE1 in the brain (left) and significant reduction of ATE1 in the retina (right) of Ate1 KO mice. Western blots were performed on brain and retina lysates from littermate pairs of wild type (WT) and KO mice. For the retina blot, WT and KO pair were on the same blot but not on the adjacent lanes. (**C**) Immunohistochemistry of central retina sections stained with anti-ATE1 (green) in WT (top) and KO (bottom), together with the G-protein G$\alpha _{o}$ (red, the marker for the ON bipolar cells) and DAPI (blue, the marker for the nuclei). The right-hand images show a 3.4x magnification of the regions of the retina shown on the left. The $+$ signs mark two representative WT and KO ON-bipolar cells, respectively. Shown here are original images of KO and WT processed at the same time with the same settings. See Fig. S1A for the corresponding images with background subtraction and/or scaling. The WT retina has clear ATE1 staining in the ONL, OPL, INL, IPL, GCL, and ON-bipolar cells; this ATE1 staining is greatly reduced in KO except bright spots that stained blood vessels (marked ‘bv’) and a few cells in INL near the border with IPL. See Fig. S1B for another example. IPL/OPL: inner/outer plexiform layer. INL/ONL: inner/outer nuclear layer. GCL: ganglion cell layer. (**D**) and (**E**), Quantification of ATE1 and G$\alpha _{o}$ levels in different retinal layers. The error bars represent SEM and the p-values are from paired Student’s t-test on 6 pairs of retinal sections from WT and KO littermates. Data points from each pair are plotted with a unique symbol.

Ate1 has been previously implicated in the functioning of the nervous system, but the effects of Ate1 knockout on neuronal responses and synaptic composition have never been investigated. To address this question, we used the mouse model in which conditional deletion of Ate1 is driven by Nestin promoter (Fig. 1A), resulting in Ate1 deletion in cells of neuronal origin during embryogenesis^16,17^. In these Ate1 knockout (KO) mice, ATE1 protein is virtually undetectable in the brain (Fig. 1B, left). These mice are weak but viable^16,17^, enabling characterization of the effects of Ate1 knockout on neuronal functions.

Previous studies have shown that Nestin-Cre is effective for gene deletion in the retina^44–47^. To test whether Ate1 was deleted from the retina of Ate1 KO mouse, we performed Western blots, and indeed, the intensity of ATE1 band was greatly decreased (Fig. 1B, right). To test ATE1 distribution in the retina, we immunostained the retina with an antibody against ATE1. In wild type (WT) retina, ATE1 staining is present in almost all layers including the outer nuclear layer (ONL), outer plexiform layer (OPL), inner nuclear layer (INL), inner plexiform layer (IPL), and ganglion cell layer (GCL) (Fig. 1C, top). In Ate1 KO, ATE1 staining was nearly absent in both the ONL and the outer tiers of INL and greatly reduced in the other layers (Fig. 1C bottom and 1D). To localize ON bipolar cells (OBCs) and to quantify their G-protein level, we co-stained the retina for ATE1 and G$\alpha _{o}$, which is a well-known marker of OBCs^48,49^. In WT retina, G$\alpha _{o}$ staining was virtually absent from ONL, and was strongest in OPL, IPL, and the cytosol of OBCs with ATE1 present in their soma, located in the outer tiers of the INL. Ate1 KO eliminated ATE1 in OBCs. It is important to notice that while ATE1 is expressed in most (or all) retinal neurons, its elimination did not change the retinal organization, the ON bipolar cells appeared normal, and G$\alpha _{o}$ distribution were indistinguishable from the wild type (Fig. 1C,E).

### Ate1 knockout mice show increased sensitivity of light-evoked responses in ON-bipolar cells

To test the role of Ate1 in retinal light responses, we next performed electroretinographs (ERG) on wild type and Ate1 KO mice. There are two types of ON-bipolar cell (OBC): the rod-OBC and the cone-OBC; both reside in the outer INL, which responds to the increments of light detected by rods and cones under scotopic and photopic conditions, respectively^48,49^. These responses elicit the scotopic and photopic ERG b-waves^50,51^.

**Figure 2:**
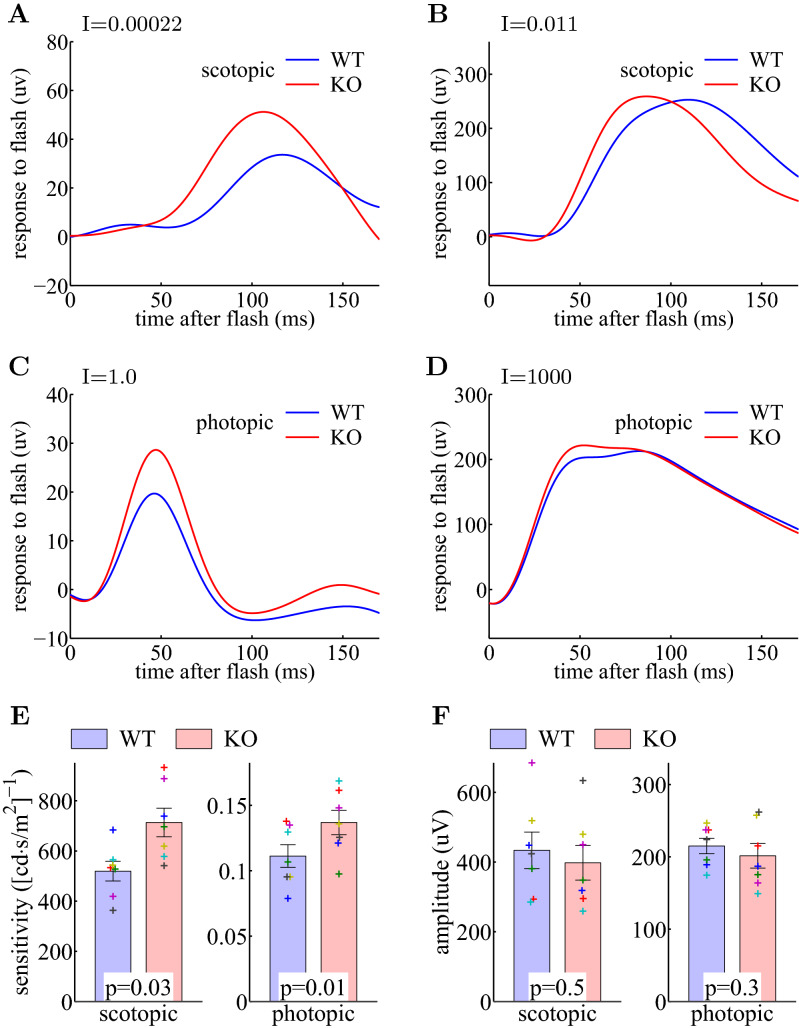
Lack of Ate1 increases the light sensitivity of ON bipolar cells. (**A**–**D**) Representative electroretinograph (ERG) responses (B-wave, 22.5 Hz low-pass filtered) of an Ate1 conditional knockout (KO, red) and a wild type (WT, blue) littermate after a prolonged dark adaptation (see "Methods"). Light flashes were delivered to the dark-adapted retina at time zero. The rod ON-bipolar sensitivity was probed with a scotopic dim flash (**A**) and a scotopic saturating flash (**B**), a 0.00022 and 0.011 $cd{\cdot }s/m^2$ green light, respectively. The cone ON-bipolar sensitivity was probed with a photopic dim flash (**C**) and a photopic saturating flash (**D**), a 1.0 $cd{\cdot }s/m^2$ green light and a 1000 $cd{\cdot }s/m^2$ Xenon light, respectively. See Fig. S2 for the representative and averaged responses to the full ranges of flashes. To probe cone ON-bipolar responses, rod ON-bipolar responses were suppressed by a constant background of 30 $cd/m^2$ green light during photopic flashes. (**E**) Quantification of the sensitivity of ERG response. The KO had significantly higher sensitivity than WT ($p=0.03$ and $p=0.01$ for rod ON-bipolar (scotopic) and cone ON-bipolar (photopic) B-wave, respectively). (**F**) Quantification of the maximum amplitude of the ERG response. The maximum amplitude did not change significantly between KO and WT. In (**E**) and (**F**), error bars represent the SEM, the p-values are from paired Student’s t-test for 7 pairs of littermates, and the data points from each pair are plotted with a unique symbol in **E** and **F**. The differences between KO and WT were also evaluated with a non-parametric statistical hypothesis test (Fig. S3), which confirmed the conclusions in (**E**) and (**F**). The B-wave sensitivity and the maximum amplitude were calculated from Eq. (1).

After prolonged (> 12 hours) dark adaptation, the ERG B-wave amplitude in response to scotopic dim light flashes (0.00022 $cd{\cdot }s/m^2$) was larger in KO than in WT (Fig. 2A), while the amplitudes for scotopic saturating flashes (0.011 $cd{\cdot}s/m^2$) were the same in both KO and WT (Fig. 2B, F, left), demonstrating a significantly higher rod-OBC sensitivity in KO retina (Fig. 2E, left). With a rod saturating background (30 $cd/m^2$), the ERG B-wave amplitude in response to photopic dim light flashes (1 $cd{\cdot }s/m^2$) was larger for KO than WT (Fig. 2C), while the amplitudes for photopic saturating flashes (1000 $cd{\cdot }s/m^2$) were the same (Fig. 2D, F, right), demonstrating a significantly higher cone-OBC sensitivity in KO retina (Fig. 2E, right). Thus, Ate1 knockout results in a gain-of-function of the sensitivity of light-evoked responses of ON-bipolar cells.

### Ate1 knockout mice show increased neuronal activity downstream of ON-bipolar cells

We next investigated the effects of Ate1 knockout on the neurons upstream and downstream of ON-bipolar cells.

**Figure 3:**
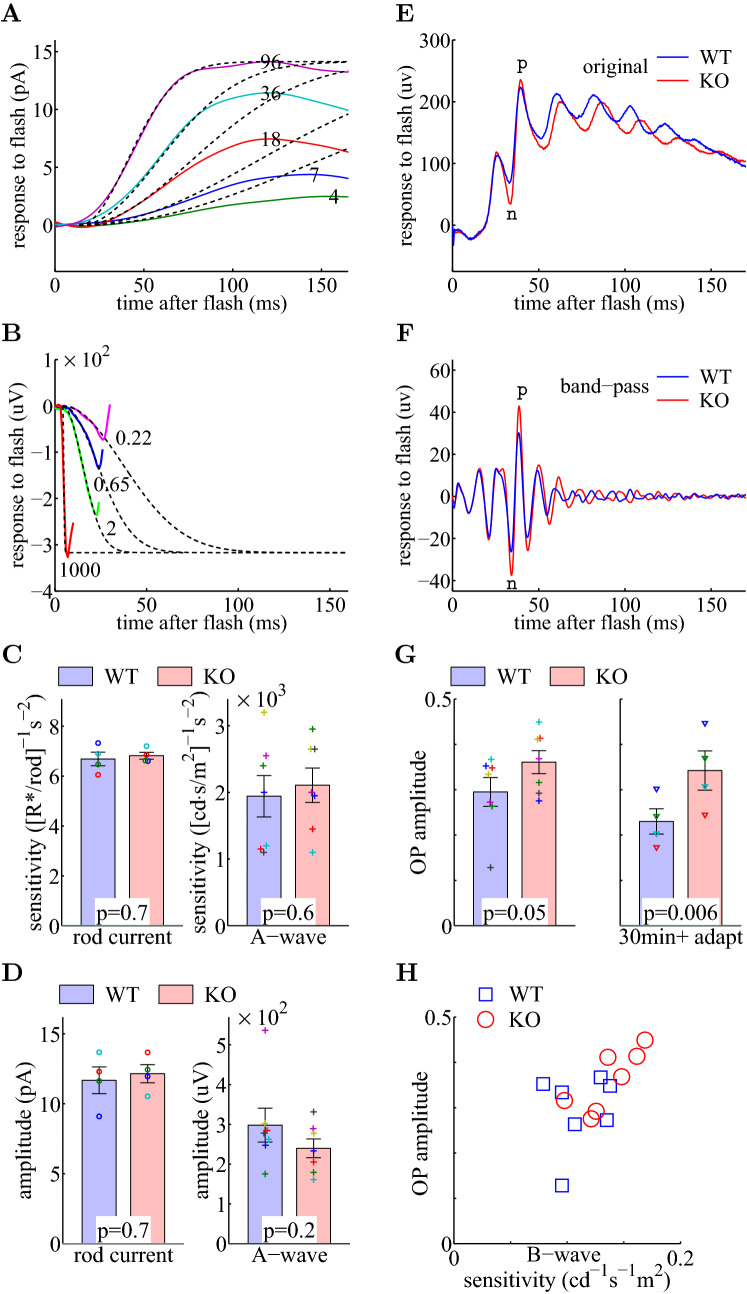
Lack of ATE1 causes no change in rod photocurrent but increases ERG oscillatory potential. (**A**) Typical Ate1 KO rod responses recorded with a suction electrode (22.5 Hz low-pass filtered) to flashes of the intensities marked in photoisomerizations (R*) per rod. The dashed lines are the best fit to Equation 2, with $S_A=7.1$ [R*/rod]$^{-1}$ $\hbox {s}^{-2}$ and $A_{max}=14.1$ *pA*. (**B**) Typical ERG A-wave responses (1K Hz low-pass filtered) to flashes of the intensities marked in $cd{\cdot }s/m^2$. The dashed lines are the best fit to Equation 2, with $S_A=2200$ [$cd{\cdot }s/m^2$]$^{-1}$ $\hbox {s}^{-2}$ and $A_{max}=317$ $\mu V$. Note: the polarity is flipped between (**A**) and (**B**). (**C**,**D**) For dark adapted mice, the sensitivities (**C**) and amplitudes (**D**) of the rod photocurrent (left) and the ERG A-wave (right) are the same for KO and WT. The error bars represent the SEM and the p-values are from paired Student’s t-test on 4 (left) and 7 (right) pairs of littermates. (**E**,**F**) Representative photopic ERG responses (**E**, the original recording; F, band-pass filtered between 75 and 300 Hz) of a KO (red) and a WT (blue) littermate pair. The timing of a pair of negative and positive peaks of the oscillatory potential (OP) were marked with n and p, respectively. The OP amplitude was defined as the difference between n and p peaks in (**F**). (**G**) The KO had significantly larger OP amplitude than WT. The y-axis shows the OP amplitude divided by the maximum amplitude of the B-wave. On the left, the raw data were collected during the same photopic recording as in Fig. 2. On the right, the mice were not dark adapted and in addition they were adapted to the 30 $cd/m^2$ rod-suppressing background for an extra 30 min. The error bars represent SEM, and the p-values are from paired Student’s t-test for 7 (left) and 4 (right) pairs of littermates. The data points from each pair are plotted with a unique symbol in (**C**), (**D**), and (**G**). (**H**) A scatter plot of the OP amplitudes versus the B-wave sensitivities demonstrates a positive correlation (Pearson/Spearman correlation coefficient=0.59/0.63; p=0.01/0.008; n=14 pairs). The corresponding data points of the OP amplitudes and the B-wave sensitivities are from Figs. 3G (left), 2E (right), respectively.

Since in Ate1 KO mice ATE1 is also eliminated in the photoreceptors in the ONL, we wanted to make sure that the sensitivity increase of ON-bipolar cells was not due to increased photo-transduction in the photoreceptors. To test this, we directly measured the photocurrent of dark-adapted rods at low illumination levels (Fig. 3A). We found that the sensitivity of the photocurrent was the same for WT and Ate1 KO (Fig. 3C, left). The scotopic ERG A-wave (Fig. 3B), dominated by rod responses^50,51^ and measured during the same experimental session as the measurements of the corresponding scotopic B-wave of dark-adapted mice, also had the same sensitivity for WT and Ate1 KO (Fig. 3C, right). We also found no significant change in response amplitudes of the photocurrent (Fig. 3D, left) or the ERG A-wave (Fig. 3D, right). In fact, the typical responses of rods from Ate1 KO (Fig. 3A) were indistinguishable from WT. Thus, the observed sensitivity increase in Ate1 KO (shown in Fig. 2E, left) did not come from rods, indicating increased sensitivity at rod-OBC synapses.

To see if Ate1 knockout has any effect downstream of ON-bipolar cells, we measured the oscillation potential (OP) riding on the rising phase of photopic ERG B-wave evoked by a saturating flash (Fig. 3E). The amplitude of the intermediate peak of OP (Fig. 3F) represents the action-potential-independent activities of neurons in the inner retina, post-synaptic to ON-bipolar cells^52^. The OP amplitude was significantly larger in Ate1 KO than WT (Fig. 3G). Since neurons in the inner retina are more sensitive to light than ON-bipolar cells^53^, we expect that they respond to the early rising phase of light-evoked ON-bipolar responses and consequently generate the OP with amplitudes related to the sensitivity of B-wave but not the saturating amplitude. It is likely that the increased ON-bipolar sensitivity caused the increased OP amplitude in Ate1 KO mice. Supporting this, the OP amplitude of Ate1 KO response was positively correlated with the corresponding B-wave sensitivity (Pearson/Spearman correlation coefficient=0.80/0.86; p=0.015/0.007; n=7 pairs; Fig. 3H).

### RGS7 and its binding partner G$\beta _5$ are enriched in the ON-bipolar dendritic tips of Ate1 knockout mice

We have shown above that G$\alpha _{o}$ in ON bipolar cells of Ate1 KO retina was unchanged (Fig. 1C, E), so G$\alpha _{o}$ is not responsbile for the increased sensitivities of light-evoked responses in OBCs. But it was important to also test whether Ate1 regulates other proteins in the glutamatergic G-protein signaling complex of OBCs.

**Figure 4:**
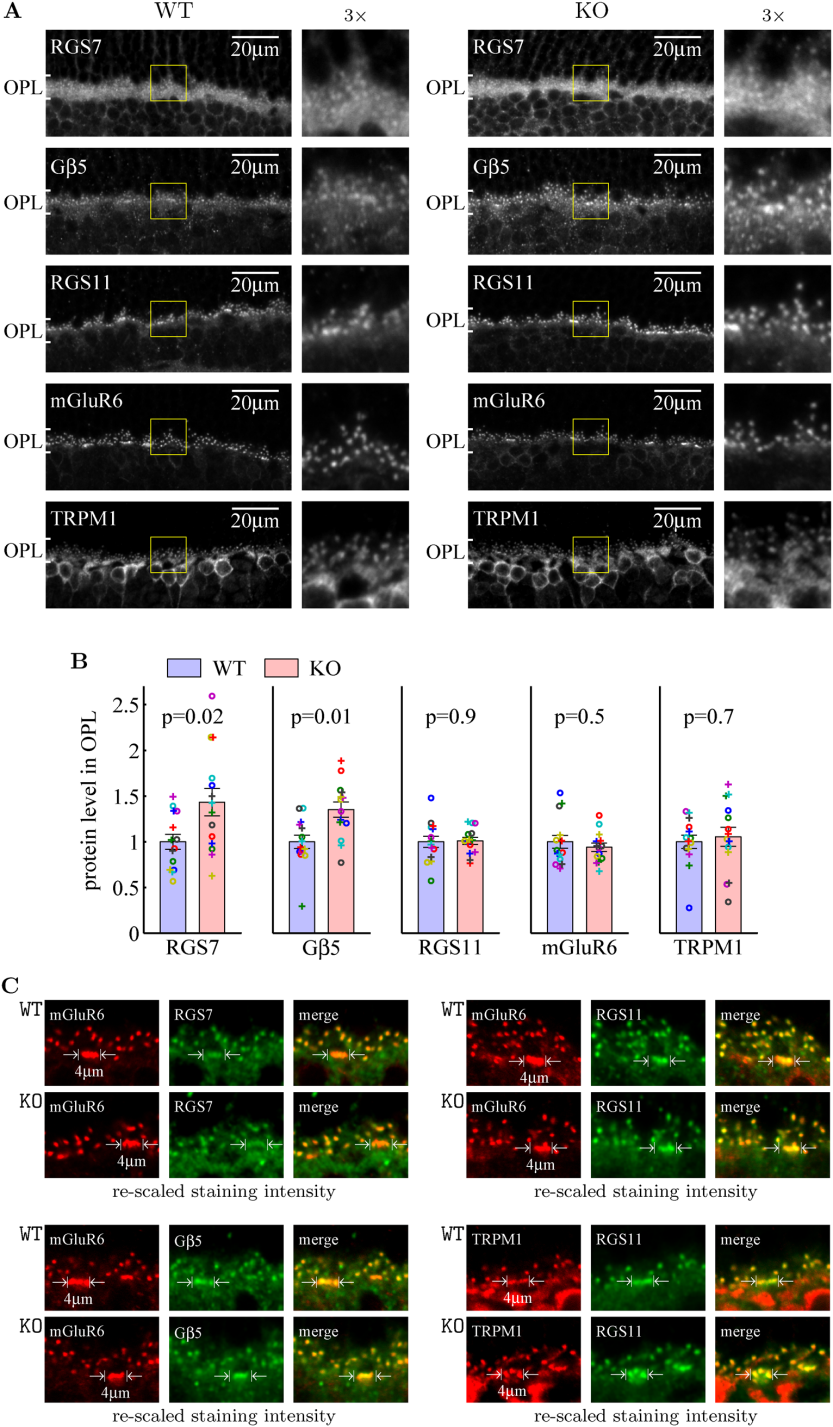
RGS7 protein and G$\beta _5$ protein are enriched in the outer plexiform layer (OPL) of Ate1 KO mice. (**A**) Immunohistochemistry of OPL in central retina stained with antibodies to RGS7, G$\beta _5$, RGS11, mGluR6, TRPM1 (top to bottom) for a representative pair of WT and KO littermates. The regions in the yellow squares are shown at 3$\times$ magnification on the right of each image. (**B**) Quantification of protein levels in the OPL measured by immunostaining intensities (see "Methods"). RGS7 and G$\beta _5$ are the only two proteins that showed significantly higher levels in Ate1 KO compared to WT. Error bars represent SEM and the p-values are from paired Student’s t-test on 14 pairs of retinal sections of 7 WT and KO littermate pairs. Data points from each pair are plotted with a unique symbol. (**C**) Various antibodies (red for mGluR6 and TRPM1, and green for RGS7, G$\beta _5$ and RGS11) co-stain individual puncta (postsynaptic to rod) as well as patches of puncta (postsynaptic to cones, between white arrows) in the OPL. Each image in (**A**) is the average over a Z-stack of cofocal images to show the staining differece between WT and KO. Each image in (**C**) is a selected section from a Z-stack to best show the co-localization, for which its intensity is re-scaled. WT and KO show no difference in co-localization.

In the dark, glutamate released by photoreceptors activates the ON-bipolar cell’s GPCR, the metabotropic glutamate receptor 6 (mGluR6) receptor, which in turn activates the $\hbox {G}_o$-proteins, causing them to close the transient receptor potential M1 (TRPM1) channel and thus hyperpolarize the OBCs; during light stimulation, the $\hbox {G}_o$-proteins in OBCs return to the inactive state and the inactive $\hbox {G}_o$-proteins enable the TRPM1 channel to open, depolarizing OBCs^54^. The light-evoked depolarization in OBC is augmented by two members of R7 family, RGS7 and RGS11^29–32^. Each of them forms a heterodimer with G$\beta _5$ and can accelerate the intrinsic GTPase activity of the G-protein by an order of magnitude^55–57^. In theory, an increased level of any component(s) of the G-protein signaling complex in Ate KO mice can lead to an increase in the sensitivity of light-evoked responses in OBCs. Using immunohistochemistry, we quantified the level of those proteins in the OPL where the signaling complex resides (Fig. 4A). Ate1 knockout had no effect on mGluR6, RGS11, and TRPM1 protein levels. In contrast, RGS7 and G$\beta _5$ signals were visibly brighter (Fig. 4A) and their levels were significantly higher in KO compared to WT (Fig. 4B).

**Figure 5:**
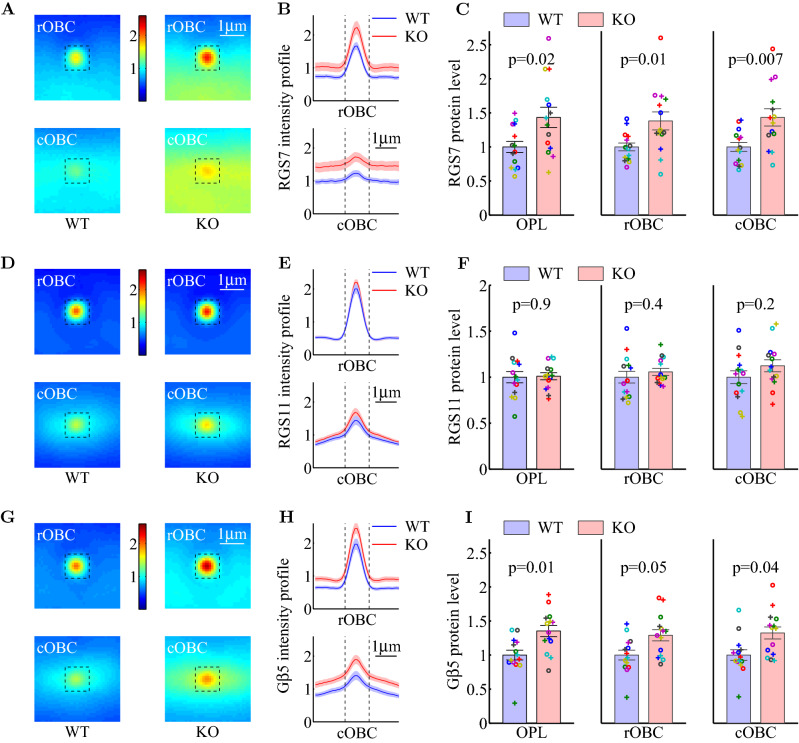
RGS7 and G$\beta _5$ protein levels specifically increase in the post-synaptic processes of Ate1 KO mice. (**A**) The average RGS7 staining intensities at all the mGluR6 staining maxima of individual puncta and in punctal patches are shown for rod-OBC (rOBC) on the top and for cone-OBC (cOBC) on the bottom, respectively. The intensity profiles over the horizontal mid-line are shown in (**B**). In (**A**) and (**B**), the background-subtracted intensity is in the unit of the background in the corresponding ONL and the dotted lines mark the central $1.1\mu m$ region surrounding punctal maxima. (**C**) The total sums at the central region show a significant increase of RGS7 proteins in the post-synaptic processes of rod-OBC and cone-OBC as well as OPL (the same as Fig. 4B, replotted here for comparison) of KO mice. (**D**–**F**) The corresponding RGS11 protein levels in the post-synaptic processes did not change in the OPL or in the dendritic tips of rod-OBC and cone-OBC. (**G**–**I**) The corresponding G$\beta _5$ protein levels also increased significantly. All averages were computed from the same 14 pairs of the retinal sections of WT and KO littermates and the data points from each pair are plotted with a unique symbol in** C**,** F**, and** I**. The p-values are from paired Student’s t-test (n=14) and the error bars represent SEM. The differences between KO and WT were also evaluated with a non-parametric statistical hypothesis test (Fig. S4), which confirmed the conclusions in (**C**), (**F**), and (**I**). For (**C**), (**F**), and (**I**), the KO/WT ratio of protein levels for individual pairs are shown in Fig. 6A and 6B.

In order for RGS7 to affect the G-protein signaling in synaptic transmission, it has to be present in the ON bipolar dendritic tips. In agreement with previous studies^30,58–61^, our immunohistochemistry staining showed that RGS7 is prominently enriched in the rod and cone ON-bipolar cell dendritic tips, which are visualized as puncta in the OPL (Fig. 4A, the magnified images) and is well co-localized with mGluR6 both in WT and KO at the rod-OBC post-synaptic density as individual puncta and at the cone-OBC post-synaptic density as elongated patches of puncta (Fig. 4C, in which the image intensities were re-scaled to better visualize the co-locolization). Simiarly, G$\beta _5$, RGS11, and TRPM1 were all well co-localized (Fig. 4C). To further test whether the over-expression of RGS7 was within these relevant postsynaptic dendritic tips, we quantified the intensity levels of the RGS7-stained puncta using the mGluR6 puncta for localization. In this quantification method, we used the total sum within the $1.1\mu m \times 1.1\mu m$ window centered at each local maximum of mGluR6 stained puncta and averaged over all the local maxima (Fig. 5A,B). Consistent with the quantification of the OPL staining, we found that the RGS11 levels in KO were similar to those in WT (Fig. 5D–F), but the RGS7 (Fig. 5A–C) and G$\beta _5$ (Fig. 5G–I) protein levels were significantly higher for KO, and this was true for both rod-OBC and cone-OBC dendritic tips.

These increases in RGS7 and G$\beta _5$ expression suggest that they contribute to the increases of ON-bipolar sensitivities in KO compared to WT observed in our ERG experiments.

### RGS7 is the driving force of increased RGS7-G$\beta _5$ levels in Ate1 knockout retinas

**Figure 6:**
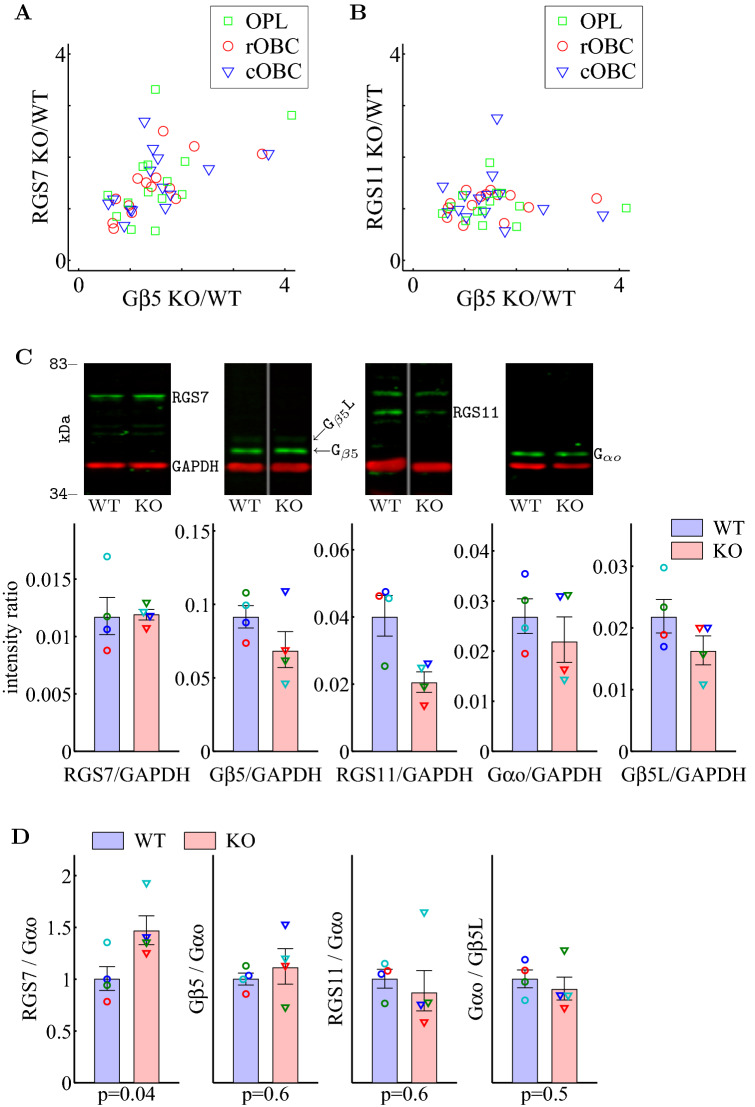
RGS7 is the driving force of increased RGS7-G$\beta _5$ levels in Ate1 knockout retinas. (**A**) A scatter plot of the paired KO/WT ratio of RGS7 versus G$\beta _5$ protein levels demonstrates a positive correlation for OPL, rOBC, and cOBC (Pearson/Spearman correlation coefficient=0.68/0.77, 0.44/0.49, and 0.54/0.55; with p-value=0.007/0.001, 0.11/0.08, and 0.04/0.05; respectively. n=14 pairs each). (**B**) A scatter plot of the KO/WT ratio of RGS11 versus G$\beta _5$ protein levels shows that there is no correlation for any of OPL, rOBC, and cOBC (Pearson/Spearman correlation coefficient=0.22/0.28, –0.10/–0.08, and 0.007/0.19; with p-value=0.44/0.33, 0.72/0.79, and 0.98/0.51; respectively. n = 14 pairs each). (**C**, top) Representative Western blots of retinal lysates showing RGS7, G$\beta _5$, RGS11, G$\alpha _{o}$, and G$\beta _5$L levels in WT and Ate1 KO retinas. (**C**, bottom) Western blot quantification of the corresponding proteins relative to GAPDH for 4 WT and 4 KO retinas from 3 litters. Data from each retina is plotted in a unique symbol. Each lane was loaded with the same amount of total proteins from each retina. For RGS7 and RGS11, each data point was the geometric average over 3 blots. (**D**) Quantification of ratios of RGS7, G$\beta _5$, and RGS11 to G$\alpha _{o}$, and G$\alpha _{o}$ to G$\beta _5$L. All ratios were normalized to WT. RGS7 ratios to G$\alpha _{o}$ increased significantly in Ate1 KO. The error bars represent SEM, the p-values are from Welch’s t-test. Paired Student’s t-test also shows the significant increase of RGS7 (Fig. S5A). To make sure that the difference of RGS7 from the others was not due to Western blot signal saturation, we performed a load test experiment which showed that both RGS7 and GAPDH signals are in the linear range (Fig. S5B).

The next question is what is the driving force of the increased RGS7-G$\beta _5$ levels in Ate1 knockout. If Ate1 regulates the protein level of either one or both of them, RGS7-G$\beta _5$ dimer level would change accordingly. However, the results of retinal immunostaining argue against G$\beta _5$ as the driving force. The bias-corrected KO/WT ratio of G$\beta _5$ levels in OPL, rOBC, and cOBC (1.34±0.04, 1.28±0.04, and 1.31±0.05) were lower than those of RGS7 (1.42±0.05, 1.38±0.04, and 1.44±0.04). Since RGS11 did not change (Fig. 5F), the apparently smaller changes of G$\beta _5$, compared to RGS7, are consistent with the fact that a significant amount of G$\beta _5$ also exists in a heterodimer form with RGS11 in the post-synaptic processes of rod- and cone-OBCs^29,62^. Consistent with this, the amount of changes of G$\beta _5$ were positively correlated with those of the RGS7 (Fig. 6A) but un-correlated with the RGS11 (Fig. 6B). Had G$\beta _5$ been the driving force, RGS11 level would also increase proportionally and the increase would also be correlated with G$\beta _5$. We suggest that Ate1 knockout facilitates the increase of RGS7 levels which in turn, drive the increase in G$\beta _5$ levels in OBC dendrites.

Next, we wished to determine if the effect of deleting Ate1 on RGS7 is true also for the retina as a whole; for that we used Western blots to examine the retinal protein levels of RGS7, G$\beta _5$, and RGS11 as well as G$\alpha _{o}$ (Fig. 6C, top). We found that in KO retina, when these protein bands are quantified relative to GAPDH, RGS7 increased slightly and the others decreased (Fig. 6C, bottom). However, GAPDH is not a good reference because it is known to be a target of Ate1 and is arginylated in vivo^63^. Instead, we used G$\alpha _{o}$. We submit that G$\alpha _{o}$ is a good reference in our case because (a) its immunostaining in WT and KO was similar (Fig. 1C,E) and (b) its levels in KO and WT, as seen by Western blots relative to G$\beta _5$ long (G$\beta _5$L, a splice variant of G$\beta _5$, also recognized by our anti-G$\beta _5$ antibody), is constant (Fig. 6C and 6D, right). G$\beta _5$L is expressed in the retina only by photoreceptors as a heterodimer with RGS9^64,65^ and is not altered or directly regulated by Ate1^4^. This quantification revealed that in the Ate1 KO mice, RGS7 protein levels was significantly higher than in WT, while G$\beta _5$ (short) and RGS11 did not change significantly (Fig. 6D). These results are consistent with the results of retinal immunostaining.

We suggest that in Ate1 knockout retina, the elevated intracellular RGS7 levels increase the stability of G$\beta _5$ by forming the heterodimer and thus driving G$\beta _5$ level up, while also out-competing RGS11 for the complex formation. Given that other R7 family proteins also form dimers with G$\beta _5$ (short) in the retina (>80% derived from RGS7+RGS11 $\sim$40%^66^ and RGS7/RGS11 $\sim$20/23^62^), it makes sense that any increase of G$\beta _5$ driven by increased RGS7 levels are proportionally smaller and therefore harder to detect.

### Proteasomal degradation of RGS7 is Ate1-dependent

To further test the hypothesis that Ate1 knockout facilitates the increase of RGS7, we examined the RGS7 expression in transfected cells which do not natively express any R7 family proteins or proteins involved in subcellular targeting of R7, including R7BP^41,42^, RGS9 anchor protein (R9AP)^67^, GPR158 or GPR179^43^, which could have impacted the RGS7 expression in the retina of Ate1 knockout mice.

It has been previously found that RGS7 levels in transfected cells are regulated by the proteasome^38^ and that some R4 family members are targeted for proteasomal degradation in Ate1-dependent manner^4^. To test whether proteasomal degradation of RGS7 is Ate1-dependent, we transfected a plasmid expressing Rgs7 into immortalized embryonic fibroblasts from wild type and Ate1 knockout mice and tested the levels of RGS7 protein in these cells in response to proteasome inhibition. We have used these cells previously to show RGS4 up-regulation by Ate1 knockout^68^. Although these cells have no endogenous expression of any R7 family protein, they do express G$\beta _5$ (short) at a fraction of the level in the brain and in the retina (Fig. S6A,B).

**Figure 7:**
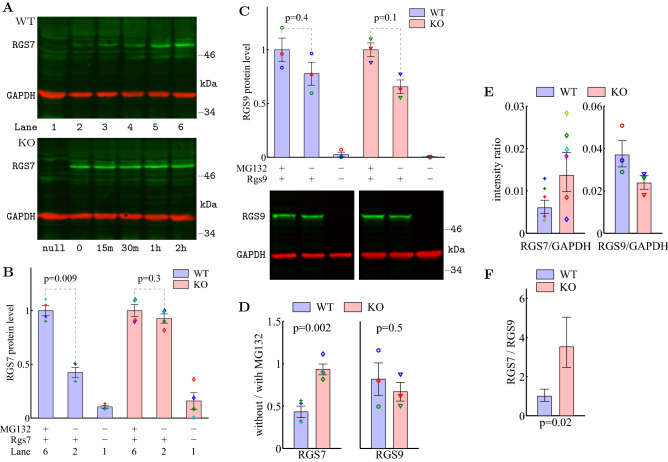
RGS7 is degraded by the proteasome in Ate1-dependent manner. (**A**) Western blot of RGS7 exogenously expressed in wild type (WT) and Ate1 knockout (KO) mouse embryonic fibroblasts transfected with Rgs7. 12 hours after transfection (lane 2), cells were further incubated with the proteasome inhibitor MG132 and were harvested at the indicated time points (lane 3 to 6). RGS7 protein level increases over time only in WT in the presence of MG132. Lane 1 in each panel shows the blot of untransfected control cells. (**B**) Western blot quantification of RGS7 in cells harvested after 2 hours of incubation with MG132, RGS7 without MG132 incubation, and RGS7 in untransfected control. RGS7 protein level significantly increased with MG132 treatment in WT, but not in KO cells. Data points from 4 independent experiments are plotted with a unique symbol for each experiment. (**C**) Western blot quantification of RGS9 in WT and KO cells from 3 independent experiments. The effect of MG132 treatment on RGS9 protein levels does not depend on the WT or KO background, indicating that its degradation is not affected by Ate1. In all colored panels of (**B** and** C**), RGS protein levels were first normalized to GAPDH loading control and then normalized for each blot. The error bars represent SEM. The p-values are from paired Student’s t-test. (**D**) The ratio of RGS7 level without/with MG132 was significantly higher in KO than WT cells while the ratio of RGS9 level without/with MG132 in KO and WT cells did not differ significantly. The error bars represent SEM. The p-values are from Welch’s t-test. (**E**) Western blot quantification of the stable RGS levels after 12 hours of transfection with GAPDH as loading control. Data from each experiment are plotted with a unique symbol. The error bars represent SEM. However, these cannot be compared directly due to the unknown factors of transfection efficiencies and GAPDH levels potentially different between WT and KO as well as antibody differences. The meaningful comparison is to compare the ratio of two RGS intensities between KO and WT, such that the factors cancel out—assuming they are constant and multiplicative. The ratios are shown in (**F**). Calculation of each average ratio was performed in log2 unit (see "Methods") and plotted in linear fashion, normalized to WT. The error bars represent STD. The p-values are from Welch’s t-test.

After 12–14 hours in culture post-transfection, RGS7 protein level became stable (Fig. 7A, lane 2; Fig. S7). In agreement with previous observations^38^, RGS7 protein levels in wild type cells increased rapidly when the transfected cells were further incubated with the proteasome inhibitor MG132 (Fig. 7A, top, lanes 3–6) and were significantly higher after 2 hours of incubation with MG132 (Fig. 7B, left). Strikingly, this effect was completely abolished in Ate1 knockout cells: no significant change of RGS7 protein level was observed during the course of incubation with MG132 (Fig. 7A, bottom, and 7B, right), suggesting that without Ate1, RGS7 does not go through proteasomal degradation. Similar to the retina, G$\beta _5$ levels did not change significantly after transfection and MG132 incubation, confirming that G$\beta _5$ is not directly regulated by Ate1 (Fig. S6C). Also similar to the retina, any increase of G$\beta _5$ due to stabilization by forming dimers with RGS7 is smaller than the increase of RGS7 because only a fraction of these cells can be successfully transfected^68^ and thus have RGS7.

As a negative control, we performed the same degradation tests on another R7 family member, RGS9, which forms heterodimer with G$\beta _5$L in photoreceptors and is known to be degraded mostly through the proteasome-independent action of lysosomal cysteine proteases^69^. As expected, MG132 incubation had no significant effect on RGS9 protein level in WT and furthermore, Ate1 knockout had no significant effect (Fig. 7C). In another comparison, the ratio of RGS7 protein level without/with MG132 was significantly higher in Ate1 KO than WT cells, while the ratio of RGS9 protein level without/with MG132 in Ate1 KO and WT cells did not differ significantly (Fig. 7D). Thus, Ate1 appears to facilitate proteasomal degradation of RGS7 but have no effect on RGS9.

To further reveal the different effects of Ate1 knockout on those RGS proteins, we quantified their stable levels after 12 hours of transfection as signal intensity relative to GAPDH (Fig. 7E). For the same reason as in the retina, GAPDH is not a good reference. Instead, we used RGS9 as the reference—since RGS9 does not appear to be affected by Ate1—and found that RGS7 was significantly higher in Ate1 KO than WT (Fig. 7F). The RGS7 change revealed in Fig. 7D and 7F is 2 to 4 fold, similar to RGS4^68^. These results further confirmed that Ate1 regulates RGS7 expression levels. The increase of RGS7 in KO relative to WT is compatible with the one we observed in the retina (Fig. 6D).

**Figure 8:**
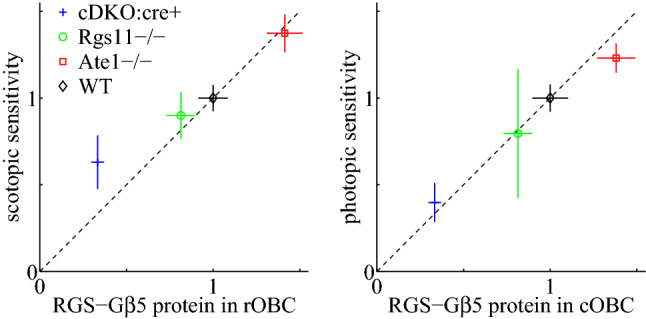
The ON bipolar light sensitivities monotonically increase with the RGS-G$\beta _5$ protein levels. The scotopic and photopic ERG sensitivities are plotted against the RGS-G$\beta _5$ protein levels in the dendritic tips of rod-OBC (rOBC) and cone-OBC (cOBC) on the left and the right, respectively. The data points for the wild type (WT) and the conditional Ate1 knockout (Ate1-/-) mice are from the current study, for which the levels of RGS-G$\beta _5$ are represented by the immunostaining levels of G$\beta _5$, the obligatory heterodimer binding partner of both RGS7 and RGS11. The data points for the RGS11 knockout (Rgs11-/-) and the tamoxifen-induced, conditional Rgs7 knockout of Rgs11-/- background (cDKO:cre+) mice are derived from^62^, for which the levels of RGS-G$\beta _5$ protein are represented by the immunostaining levels of RGS7 (no RGS11). The cDKO:cre+ data point is from mice with 12 days of tamoxifen administration (RGS7 level was significantly reduced but not completely eliminated yet). All the values are normalized to the corresponding ones of WT. The cDKO:cre+ values (at day 12) are first normalized by the corresponding ones at day 0, and then normalized with the Rgs11-/- together by the WT values from^62^. The normalized level of RGS protein in Rgs11-/- is estimated to be 35/43. This is derived from 20 RGS7 and 23 RGS11 (fmol/10mg) in WT, and 35 RGS7 (fmol/10mg) in Rgs11-/-^62^. The ratio of 35 to 20 RGS7 in Rgs11-/- versus WT is also consistent with^30^.

**Figure 9:**
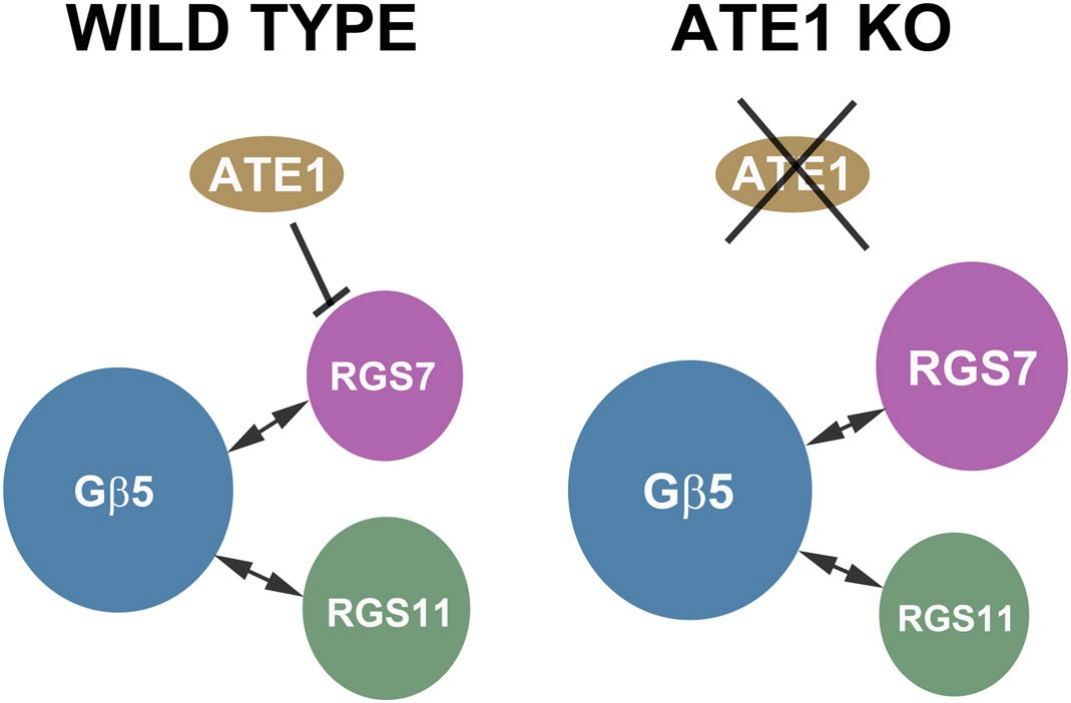
An interaction model illustrates how RGS7 degradation is Ate1-dependent and Ate1 knockout increases the level of RGS7. Since RGS7 competes with RGS11 (or other R7 RGS proteins) for binding with G$\beta _5$ (or other R7 binding proteins) and increases the stability of G$\beta _5$ through dimer formation, an increase in RGS7 can cause a decrease in RGS11 and/or an increase in G$\beta _5$.

**Figure 10:**
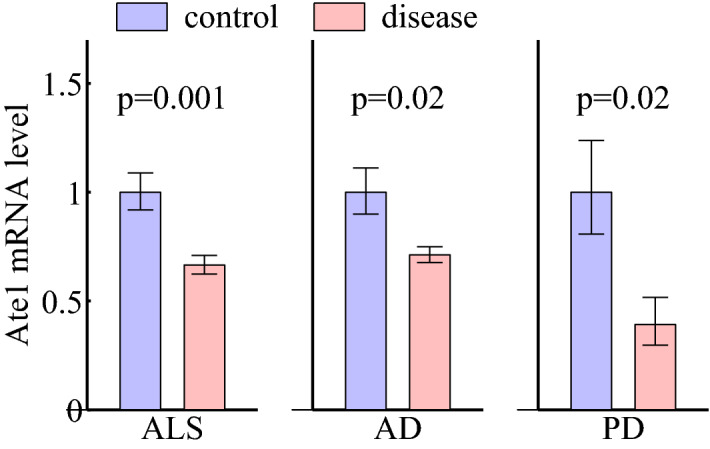
Levels of Ate1 mRNA are significantly lower in several types of neurodegenerative diseases. ALS: amyotrophic lateral sclerosis, comparing mouse microglia sorted from whole spinal cord of control and late stage TDP-43 proteinopathy, calculated from the public GSE109171 RNAseq data set^75^ normalized to $\beta$-actin. AD: Alzheimer’s disease, comparing laser captured human hippocampal gray matter of normal brain and severe AD patients, calculated from the public GSE28146 microarray data set^76^ using RMA method. PD: Parkinson’s disease, comparing laser-captured human dopaminergic neurons in substantia nigra of normal brain and PD patients, calculated from the public GSE20141 microarray data set^77^ using RMA method. Plot showing relative Ate1 mRNA expression levels. Calculation was performed in log2 unit and plotted in linear fashion. The error bars represent SEM and the p-values are from Welch’s t-test.

## Discussion

Our study demonstrates that RGS7, which plays a key role in G-protein signaling during neuronal responses, is regulated by arginyltransferase 1 (Ate1), an essential enzyme that mediates a post-translational modification of global biological importance. We show that intracellular levels of RGS7 depend on the presence of Ate1, on which the proteasomal degradation of RGS7 depends, and that impairments in this mechanism lead to increased RGS7 in the nervous system and altered responses in neurons. This (1) gives a new perspective on how G-protein signaling is modulated by different levels of RGS proteins, (2) demonstrates a new pathway of RGS7 regulation, and (3) implicates a wider role of Ate1 in neuronal functions and diseases.

First, the present study shows that an increased RGS7 level in the retina of Ate1 KO mice (Figs. 4A,B, 5 A–C) is accompanied by an increase of the ON-bipolar sensitivities (Fig. 2E). This is consistent with the previous study of the tamoxifen-induced, conditional Rgs7 knockout mice of Rgs11-/- background (an Rgs11 knockout mice with tamoxifen-induced, conditional Rgs7 knockout), which showed that when RGS7 level was reduced progressively the ON-bipolar sensitivities were reduced accordingly^62^. However, Ate1 knockout did not change the protein levels of G$\alpha _{o}$, mGluR6, and TRPM1 of the G-protein signaling complex in OBC dendrites in the OPL (Figs. 1C,E, 4 A, B) and did not change the saturating ON-bipolar response amplitudes (Fig. 2F). This is consistent with the fact that in dark adapted retinas, these amplitudes are limited by the number of TRPM1 channels opened by the signaling cascade, which are saturated in wild type—in such a case, variation in the RGS levels, even the complete elimination of either RGS7 or RGS11 alone, does not substantially change the amplitudes^29–31^. The increased RGS7 together with its binding partner G$\beta _5$ in the dendritic tips of Ate1 KO OBCs mostly increases the small signal responses—and thus the sensitivities of OBCs (Fig. 2A,C,E) and the activities downstream of OBCs (Fig. 3E–H). Although not statistically significant, we did observe an acceleration on the rising phase of ON-bipolar responses in Ate1 KO (e.g., Fig. 2A,B), also expected to occur with increased RGS7 levels.

To look at the relationship between ON-bipolar sensitivities and RGS-G$\beta _5$ protein levels from a broader perspective, we combine data from previous studies^62^ with that of the current study, and show that the scotopic and photopic sensitivities monotonically increase with the RGS-G$\beta _5$ protein levels in the dendritic tips of rOBC and cOBC, respectively (Fig. 8). Therefore, the high amount of RGS proteins in OBCs—by some estimation at a three-to-one ratio to the GPCR (mGluR6) dimer^62^—is not redundant or excessive; it does not change the amplitude of ON-bipolar responses, but rather boosts their sensitivity, which is a behaviorally important function. This is significant because the downstream neurons in the inner retina also respond to the rising phase of ON-bipolar responses and reach response peaks well before OBCs (^53^ and Fig. 3E, F).

Second, the present study shows that ATE1 facilitates the proteasomal degradation of RGS7 and Ate1 knockout leads to increased RGS7 levels in vivo. This is in contrast to all previously known proteins which regulate RGS7 expression. All those proteins stabilize RGS7 and impede its degradation.

Because a small number of different members of the RGS family are expressed in various brain regions to regulate a very large number of GPCR signaling pathways^25^, precise control over the intracellular levels of each RGS protein is very important in modulating the complexity of these pathways. Regulation of RGS protein levels can be partially achieved through differential transcription and/or translation of RGS in different brain regions under different physiological conditions^70^. However, the observed discrepancies between Rgs mRNA levels^71^ and RGS proteins levels^72^ point to additional mechanisms that regulate the amount of each RGS protein post-translationally by differential degradation or stabilization of RGS proteins. Our study demonstrates that degradation of RGS7 is facilitated by Ate1. Without Ate1, the main RGS7 degradation pathway through proteasome stops working (Fig. 7A,B) and RGS7 protein levels increase in MEF cells (Fig. 7F) and in the retina (Fig. 6D). In MEF cells, this role of Ate1 is independent of R7BP, GPR158, and GPR179 because they are not expressed. In the retina, this role of Ate1 is independent of G$\alpha _{o}$ since there is no significant difference between KO and WT from the immunostaining (Fig. 1C,E) and the Western blot (Fig. 6C,D) of G$\alpha _{o}$. This role of Ate1 is also independent of Pkd1 and Tnf because there is no significant expression of Pkd1 and Tnf in either the retina^73^ or MEF cells, and we also did not observe any change of their expression due to Ate1 knockout. Of all the proteins which are known to stabilize RGS7, only G$\beta _5$ levels are dependent on Ate1 but to a lesser extent than RGS7 (Figs. 5C, I, 6D). We propose that Ate1 KO leads to the increased stability of RGS7 and thus facilitates the increase of RGS7-G$\beta _5$ dimer and consequently higher G$\beta _5$ level and we further suggest that the increased RGS7 out-competes RGS11 in forming dimer with G$\beta _5$ and thus causes RGS11 level to decrease (Fig. 9).

Third, the present study shows that RGS7 is regulated differently from RGS9 and RGS11, the two proteins that belong to the same R7 family and are co-expressed with RGS7 in certain neurons. Like bipolar cells in the retina with similar molar amounts of RGS7 and RGS11^62^, striatal neurons in basal ganglia contain equal levels of RGS7 and RGS9 that show different subcellular distribution^74^. Notably, both RGS9 and RGS11 are metabolically less stable than RGS7 and they both require other binding partners—in addition to G$\beta _5$—to stabilize them^57,69^. This difference in stability, and the mechanisms regulating these proteins in vivo, could be beneficial for neural adaptation and synaptic remodeling that happens on different time scales and/or with different physiological causes. On the other hand, because R7 family members compete for the same pool of binding partners, regulating one can affects others^74^. Since RGS7 has higher GTP hydrolysis rate than RGS9 and RGS11^57^, RGS7 substituting just a portion of RGS9 or RGS11 in their cohabited neurons can further accelerate the de-activation of G-protein signaling. As a result, in addition to RGS7, Ate1 is expected to affect functions otherwise dominated by other R7 family members.

We previously showed that the Ate1 knockout mice used in this study developed symptoms of neurodegeneration^17^. Our present study expands the list of RGS proteins regulated by Ate1 from R4 family to R7 family and opens the possibility that Ate1 regulates functions of many RGS proteins, which are implicated in neurodegeneration including amyotrophic lateral sclerosis, Parkinson’s and Alzheimer’s diseases^26^. Intriguingly, high throughput studies from other groups show that Ate1 is significantly reduced in several of these neurodegenerative diseases (Fig. 10). Although it is tempting to attribute the Ate1 connection to neurodegeneration to its direct interaction with the proteins which aggregate in those diseases^17,78,79^, it is prudent to ask whether Ate1 also plays an independent role there through regulating RGS proteins and inducing neuronal dysfunctions as shown in our present study. Since RGS7, unlike the Ate1 targets from the R4 family, does not possess an N-terminal sequence suitable for the N-degron, it appears likely that this degradation, in the case of RGS7, is mediated via another pathway—e.g., by modification of the acidic side chains of the midchain Asp or Glu residue(s), previously demonstrated to occur on ATE1 protein targets in vivo^80^. It is also possible that in the case of RGS7, its Ate1-dependent degradation occurs by another, as yet unknown mechanism. Answering these questions constitute exciting directions of future studies.

## Methods

### Ethical approval and accordance

Procedures involving animals were performed in accordance with National Institute of Health guidelines and the protocol was reviewed and approved by the Institutional Animal Care and Use Committee of the University of Pennsylvania. This study was carried out in compliance with the ARRIVE guidelines (http://www.nc3rs.org.uk/page.asp?id=1357). The total number of animals used in the present study was 18 wild-type (WT) mice and 17 Ate1 conditional knockout (KO) mice. The earliest age used for the experiments was 8 weeks old; both male and female mice were used. When possible, pairs of WT and KO littermates were used in each experiment and processed in parallel; otherwise, WT and KO of each pair/litter were processed close in time and their order were randomized for different pairs/litters. When possible, each animal was used in multiple experiments and data were collected from both eyes.

### Generation of Ate1 conditional knockout mice

Ate1-floxed mice^81,82^ were crossed with commercially available mouse line expressing Cre recombinase under Nestin promoter (Jackson Laboratory strain B6.Cg-Tg(Nes-cre)1Kln/J). Mice were bred and maintained in a mixed C57BL6/129SVJ background according to approved animal protocols.

### Western blotting

For brain and retina, freshly excised mouse tissues were flash-frozen in liquid nitrogen and homogenized by grinding using mortar and pestle in liquid nitrogen, then resuspended in SDS sample buffer, fractionated by SDS-PAGE and transferred onto nitrocellulose membranes for Western blotting^16^. For transfected cells, after washing with PBS on ice, cells were collected into Eppendorf tubes and centrifuged at 17000g for 5 min, then PBS was removed and the pellets were weighted and resuspended in 1:10 (w/v) of 2$\times$SDS buffer, heated to 95$^o$C for 15 min, then centrifuged at 17000g for 15 min. The supernatants were diluted with 1$\times$SDS buffer and loaded onto 12% SDS-PAGE for Western blot. Protein bands were detected using the Odyssey CLx infrared scanning system and quantified with Image Studio Lite Ver3.1 (Li Cor, Lincoln, NE, USA). Signal intensity was normalized to GAPDH signal for comparison of protein levels between samples.

### Immunofluorescence of retinal cryosections

Mice were euthanized with a mixture containing ketamine/xylazine ($300\mu \hbox {g}$ each per g body-weight). Immediately after the euthanasia, eyes were enucleated and the cornea removed. Eyeballs were fixed in 4% paraformaldehyde for 60 minutes, rinsed in phosphate buffer (PB), cryoprotected overnight at 4$^o$C in 0.1M PB containing 30% sucrose and embedded in a mixture of two parts 20% sucrose in PB and one part optimal cutting temperature compound (Tissue Tek, Electron Microscopy Sciences, Hateld, PA, USA). Radial sections ($16\mu \hbox {m}$) were cut on a cryostat (Leica Biosystems, Buffalo Grove, IL, USA) and were collected on a superfrost plus glass slide (Fisher Scientific, Pittsburgh, PA, USA). Retinal cryosections were permeabilized and blocked in 0.1M PB containing 5% sucrose, 10% normal goat serum or normal donkey serum, and 0.5% Triton X-100 for 30-45 minutes at room temperature. Sections were incubated with primary antibodies diluted in the blocking buffer at room temperature for 2 hours, followed by three 10 minute washes with 0.1M PB containing 5% sucrose. Sections were then incubated with secondary antibodies diluted in the blocking buffer at room temperature for 1 hour, followed by three 10 minute washes with 0.1M PB containing 5% sucrose. Finally the slides were mounted using Vectashield mounting medium (Vector Laboratories, Burlingame, CA, USA) and imaged using a confocal laser scanning microscope (Olympus Fluoview 1000, Center Valley, PA, USA) under an oil-immersion objective^83^. Littermate retinas from WT and KO mice were immunostained and imaged in parallel under the same conditions and settings. Each pair of retinas from WT and KO littermates were also collected and processed together. To compensate the exposure difference between imaging of retinal slides, each set of images from the same retinal slide was normalized by the non-specific signal in the the outer nuclear layer (ONL).

### Immunostaining image processing

A region of interest (ROI, of the same $105\times 7.5\mu \mathrm{m}$ area size for WT and KO mice) was drawn around the outer plexiform layer (OPL), and the intensity measurement for each pixel was taken from the average z-stacks (of the same $0.3\mu \hbox {m}$/slice thickness for WT and KO mice) using MATLAB software (MathWorks, Natick, MA, USA). The pixel intensity was thresholded by the background—the mean pixel intensity from the ONL. The average intensity of the pixels in the ROI was calculated for each sample to represent the immunostaining level in OPL, with 2 samples per retina (one superior and one inferior of central retina). We used mGluR6 as a marker to locate the ROI in OPL and to find the centers of the dendritic tips of ON-bipolar cells (OBCs). We located all the local maxima in the three dimensional volume of ROI and z-stacks, which was further selected by a two dimensional mask for potential OBC dendritic tip areas (from the averaged z-stacks) to get the visually confirmed OBC dendritic tips. To discriminate between rod-OBCs and cone-OBCs, we used the criteria that for a rod-OBC dendritic tip the masked area extends no more than $0.5\mu \hbox {m}$ on each side of the center, while for a cone-OBC dendritic tip the masked area extends more than $1.1\mu \hbox {m}$ including the center.

### Electroretinograph

The electroretinograph (ERG) recording were performed as described previously^50,51^. Mice were dark adapted for more than 12 hours in their cage (with food and water), which was placed inside a specially aerated black box. Before ERG, mice were deeply anesthetized under dim red light by intraperitoneal injection of a mixture containing ketamine/xylazine/urethane ($20\mu \hbox {g}$/$8\mu \hbox {g}$/$800\mu \hbox {g}$ per g body-weight) and were placed on a platform maintained at 38$^o$C. Pupils were dilated with 1% tropicamide saline solution (Mydriacyl, Alconox, New York, NY, USA). A platinum electrode was inserted into the mouth to serve as the reference and the ground electrode, another platinum electrode was placed on the cornea of each eye, and the platform was moved inside a light-proof Ganzfeld Faraday cage during ERG. ERG recordings from KO and their WT littermates were performed on the same day under the same settings and conditions. Light stimuli were either 4 ms flashes produced by a green light-emitting diode (LED) or <1 ms flashes produced by a Xenon tube delivered in the Ganzfeld (Espion Electrophysiology System; Diagnosys, Lowell, MA, USA). For photopic stimuli, a rod-suppressing background (green LED light, 30 scotopic $cd/m^2$) was given and light flashes were superimposed on it 3 to 6 minutes after the onset of the background. ERGs were recorded from both eyes using differential amplifiers with a bandwidth of 0.1 Hz to 1 kHz, and were sampled at 0.2 ms intervals. Depending on the signal-to-noise ratio, a typical recording contained an average of 3 to 20 individual responses. At the end of the recording session, animals were removed from the platform, returned to the cage, observed until they fully recovered, and then brought back to the facility. These animals were checked daily and either euthanized within a week to obtain their retinas, or kept alive for several more weeks before an additional ERG recording session followed by euthanasia.

### Suction pipette recording from rods

The rod outer-segment-in technique for photocurrent recording was described in^84^. Before each experiment, mice were dark adapted for more than 12 hours in their cage (with food and water), which was placed inside a specially aerated black box. Mice were intraperitoneally injected with a lethal dose of ketamine/xylazine mixture ($300\mu \hbox {g}$ each per g body-weight) under dim red light. Eyes were quickly enucleated and dissected under infrared light in a Petri dish filled with Lockes solution (112.5 mM NaCl, 3.6 mM KCl, 2.4 mM $\hbox {MgCl}_2$, 1.2 mM $\hbox {CaCl}_2$, 10 mM HEPES, 0.02 mM EDTA, 20 mM $\hbox {NaHCO}_3$, 3 mM $\hbox {Na}_2$-succinate, 0.5 mM Na-glutamate, 10 mM glucose) and bovine serum albumin (BSA; 0.1 mg/ml). Small pieces of retina were excised, sliced, and transferred to a recording chamber. The chamber was perfused with Lockes solution (pH 7.4, no BSA added) and maintained at 35-37$^o$C with a heating system designed for microscopy (Warner Instruments, Hamden, CT, USA). The Lockes solution was continuously bubbled with a gas mixture containing 95% $\hbox {O}_2$/5% $\hbox {CO}_2$ before it was directed into the chamber. Freshly pulled glass pipettes with a tip internal diameter of 2 $\mu$m were used to suck a rod outer segment in for recording. The pipette, pipette holder, and a tube connecting pipette holder to the pressure control system were filled with Lockes solution. A small air bubble separated Lockes solution from the oil-filled pressure control system. Responses were evoked with flashes of 500 nm green LED light. Light intensities were measured with a calibrated photodiode (OSI Optoelectronics, Hawthorne, CA, USA). The light intensity was converted to photoisomerizations per rod using 0.5 $\mu$ $\hbox {m}^2$ collecting area per rod. All responses were recorded with a differential amplifier with a bandwidth of 0–50 Hz and were sampled at 1 ms intervals.

### Analysis of response sensitivities

The ERG B-wave response sensitivity $S_B$ and the maximum amplitude $B_{max}$ were calculated by fitting the B-wave amplitudes in response to two light flashes of different intensities using the following equation:

1 $$\begin{aligned} B/B_{max}={S_BI/(1+S_BI)} \end{aligned}$$

in which *B* is the amplitude of B-wave response to a light flash of intensity *I* (note: $S_B$ is the inverse of half saturating intensity, $I_{0.5}$, e.g., in^62^). The values of $S_B$ and $B_{max}$ were averaged over the two eyes for each mouse.

The ERG A-wave sensitivity $S_A$ and the maximum amplitude $A_{max}$ were calculated by fitting A-wave rising phase time courses, *A*(*I*, *t*), using the equation of^85^:

2 $$\begin{aligned} A(I,t)/A_{max}=1-exp(-S_AI(t-t_d)^2) \end{aligned}$$

in which $t_d$ is a constant time delay. For each flash of intensity *I*, the rising phase of the corresponding A-wave was chosen to be the time period from $t_d$ after the light flash until reaching the maximum (or 5 msec before—if the period is longer than 5 msec). The value of $t_d$ was fitted for each eye, varying between 3.4 to 4.4 msec. The values of $S_A$ and $A_{max}$ were averaged over the two eyes for each mouse.

The rod photocurrent sensitivity was calculated by fitting the same equation as ERG A-wave, except that the rising phase was chosen to be fixed at 60 msec (flashes used in these experiments were much dimmer than ERG) and the value of $t_d$ was fitted for each rod, varying between 14 to 17 msec due to small variations of capacitance and/or conductance between the rods and the suction pipettes. The values of $S_A$ and $A_{max}$ were averaged over the 3 to 6 recorded rods for each mouse.

For the numerical analysis, the original response data were digitally filtered by a 6th order Butterworth filter with a cutoff frequency of 22.5*Hz*, 1*kHz*, and 22.5*Hz* to remove high frequency components in B-wave, A-wave, and rod photocurrent, respectively.

### mRNA analysis

Original .CEL files of microarray public data sets (GSE28164 and GSE20141 generated from Affymetrix HG-U133_Plus_2) were processed by Affymetrix Expression Console using Probeset-Summarize-Engine with default setting of the robust multi-array average (RMA^86^) method to calculate the expression level for each probeset of the array.

RNA sequencing (stranded TruSeq with Ribo-Zero, Illumina, San Diego, CA, USA) was used to quantify mRNA levels in whole brain lysates. WT and KO brain lysates from 3 littermate pairs of 3 month old mice were used for RNA sequencing with $\sim$ 100 million (50 million paired-end) reads per sample. RNA sequencing (non-stranded DNBSeq with Oligo dT, BGI, Cambridge, MA, USA) was used to quantify mRNA levels in cell lysates. WT and KO cell lysates from 4 MEF pairs were used for RNA sequencing with $\sim$ 50 million (25 million paired-end) reads per sample. Using STAR software package^87^, the reads were aligned to all mouse chromosomes plus mouse mitochondrial DNA (GRCm38). For each sample, the total number of uniquely matched fragments (paired reads) to any exon of a gene was counted as the mRNA expression level of the gene in that sample expressed in Fragments Per Kilobase of transcript per Million mapped reads (FPKM) for each gene’s relative expression.

### Cell transfection and inhibitor treatment

Rgs (7 or 9) plasmids were transfected into immortalized wild type and Ate1 knockout mouse embryonic fibroblasts^8^ using Lipofectamine®-2000 Transfection Reagent (Life Technologies). After 8-16 hrs in culture, cells were split into equal dishes, grown overnight, and then incubated either in the Dulbecco’s modified Eagle’s medium alone or in the presence of $20\mu \hbox {M}$ MG132 (Sigma, St. Louis, MO, USA). Untransfected cells were processed in parallel.

### Antibodies and constructs

Rat monoclonal ATE1 antibody (EMD Millipore, MABS436) as previously described^88^; Mouse monoclonal antibody [6C5] against GAPDH (dilution: 1:10000 for Western blotting) was purchased from Abcam (ab8245); sheep anti-mGluR6 (dilution 1:400), sheep anti-TRPM1 (dilution 1:400), as well as the Rgs7 and Rgs9 expression plasmids were the gifts from Dr. K.A. Martemyanov, The Scripps Research Institute, Jupiter, FL, USA; rabbit anti-G$\alpha _o$ (dilution 1:500; 1:2000 for Western blotting) was a gift from Dr. D. Manning, University of Pennsylvania, Phildadelphia, PA, USA; rabbit anti-G$\beta$5 (dilution 1:500; 1:1000 for Western blotting) was a gift from Dr. C.K. Chen, Baylor College of Medicine, Houston, TX, USA; rabbit anti-RGS7 (dilution 1:100; 1:1000 for Western blotting) and rabbit anti-RGS11 (dilution 1:1000) were the gifts from Dr. T.G. Wensel, Baylor College of Medicine, Houston, TX, USA.

### Experimental design and statistical analysis

Most of the experiments were performed in KO and WT littermate pairs, for which the p-values were calculated from paired Student’s t-test on the *n*-number of experiments of KO/WT pairs. For Pearson/Spearman correlation coefficients, the p-values were calculated by transforming the correlation between *n* pairs of concerned variables to create a t-statistic having $n-2$ degrees of freedom. Everything was computed using MATLAB software.

For both Western blotting and immunostaining of brain and retina tissues, the relative protein level, the KO/WT ratio, was estimated from paired samples. Each sample of WT and KO pair was always stained and imaged together. Pre-processing of the data was done to reduce the potential experimental variation: the average staining intensity of all samples on each blot or slide was normalized to 1 and then the average staining intensity of all samples of each WT and KO pair was normalized to 1.

A direct estimate for KO/WT ratio would be

$$\begin{aligned} r={\sum _{i=1}^n y_i \over \sum _{i=1}^n x_i}\ \ \ \ \hbox {and}\ \ \ \ s^2={1\over n}\sum _{i=1}^n(r_i-r)^2 \ \ \ \hbox {with}\ \ \ \ r_i={\sum _{j\ne i}^n y_j \over \sum _{j\ne i}^n x_j} \end{aligned}$$

in which $(x_i,y_i)$ are the staining intensities of a WT and KO sample pair ($i=1\ldots n$) and *s* is a Jackknife estimation of the standard deviation of the ratio *r*. However, this is a biased estimation. We used the following correction for the estimation of the ratio

$$\begin{aligned} r_{c}=nr - {n-1\over n}\sum _{i=1}^n r_i \end{aligned}$$

which reduces the bias to $O(n^{-2})$ or less^89,90^ and for the estimation of the standard deviation

$$\begin{aligned} s_c^2={1\over n-1}\sum _{i=1}^n(r_i-r_{c})^2 \end{aligned}$$

Similarly, for transfected cells, the relative protein level was estimated as the ratio of the staining intensity without MG132 to the staining intensity with MG132.

For experiments not performed in pairs or when there was a possibility that the two conditions might have different variances, the p-values were calculated from Welch’s t-test by

$$\begin{aligned} I_{v\over {v+t^2}}({v\over 2},{1\over 2}) \ \ \ \ \hbox {with}\ \ \ t= { {a_1 - a_2}\over {\sqrt{s_1^2 + s_2^2}} } \ \ \ \ \hbox {and}\ \ \ v \approx {(s_1^2+s_2^2)^2\over {{s_1^4\over v_1-1}+{s_2^4\over v_2-1}}} \end{aligned}$$

where *I* is the regularized incomplete beta function with the statistic *t* and the degrees of freedom *v* in which $a_1$, $s_1$, and $v_1$ are the mean, the SEM, and the size of the 1$^{st}$ sample, and $a_2$, $s_2$, and $v_2$ of the 2$^{nd}$ sample. To estimate the difference between WT and KO ratios of RGS7 and RGS9 proteins, we applied the estimation twice, so that

$$\displaystyle t= {(a_1 - a_2)-(a_3-a_4)\over {\sqrt{s_1^2 + s_2^2+s_3^2+s_4^2}} } \ \ \ \ \hbox {and}\ \ \ v \approx {(s_1^2+s_2^2+s_3^2+s_4^2)^2\over {{s_1^4\over v_1-1}+{s_2^4\over v_2-1}+{s_3^4\over v_3-1}+{s_4^4\over v_4-1}}}$$

in which the 1$^{st}$ and the 2$^{nd}$ samples are WT RGS7 and WT RGS9 while the 3$^{rd}$ and 4$^{th}$ samples are KO RGS7 and KO RGS9, with all samples in *log*() expression.

## Supplementary information

Supplementary information.

## Data Availability

The datasets generated during and/or analysed during the current study are available from the corresponding author on reasonable request.
