# Supplementary material for: Arginyltransferase (Ate1) regulates the RGS7 protein level and the sensitivity of light-evoked ON-bipolar responses
Source: Sci Rep. 2021 Apr 30;11:9376. doi: 10.1038/s41598-021-88628-3 (PMC8087773; doi:10.1038/s41598-021-88628-3)
Supplement: Supplementary file 1 — Supplementary information. [file 41598_2021_88628_MOESM1_ESM.pdf]

Supplementary Material:

Arginyltransferase (Ate1) regulates the RGS7 protein level and the sensitivity of light-evoked ON-bipolar responses

Marie E. Fina[a], Junling Wang[a], Sergei S. Nikonov[b], Stephanie Sterling[a], Noga Vardi[b], Anna Kashina*[a], Dawei W. Dong*[a,c]

[a] *Department of Biomedical Sciences, School of Veterinary Medicines; University of Pennsylvania, Philadelphia, PA 19104*
[b] *Department of Neuroscience, Perelman School of Medicine; University of Pennsylvania, Philadelphia, PA 19104*
[c] *Institute for Biomedical Informatics, Perelman School of Medicine; University of Pennsylvania, Philadelphia, PA 19104*

---

* Corresponding authors' email addresses: akashina@upenn.edu, ddong@upenn.edu

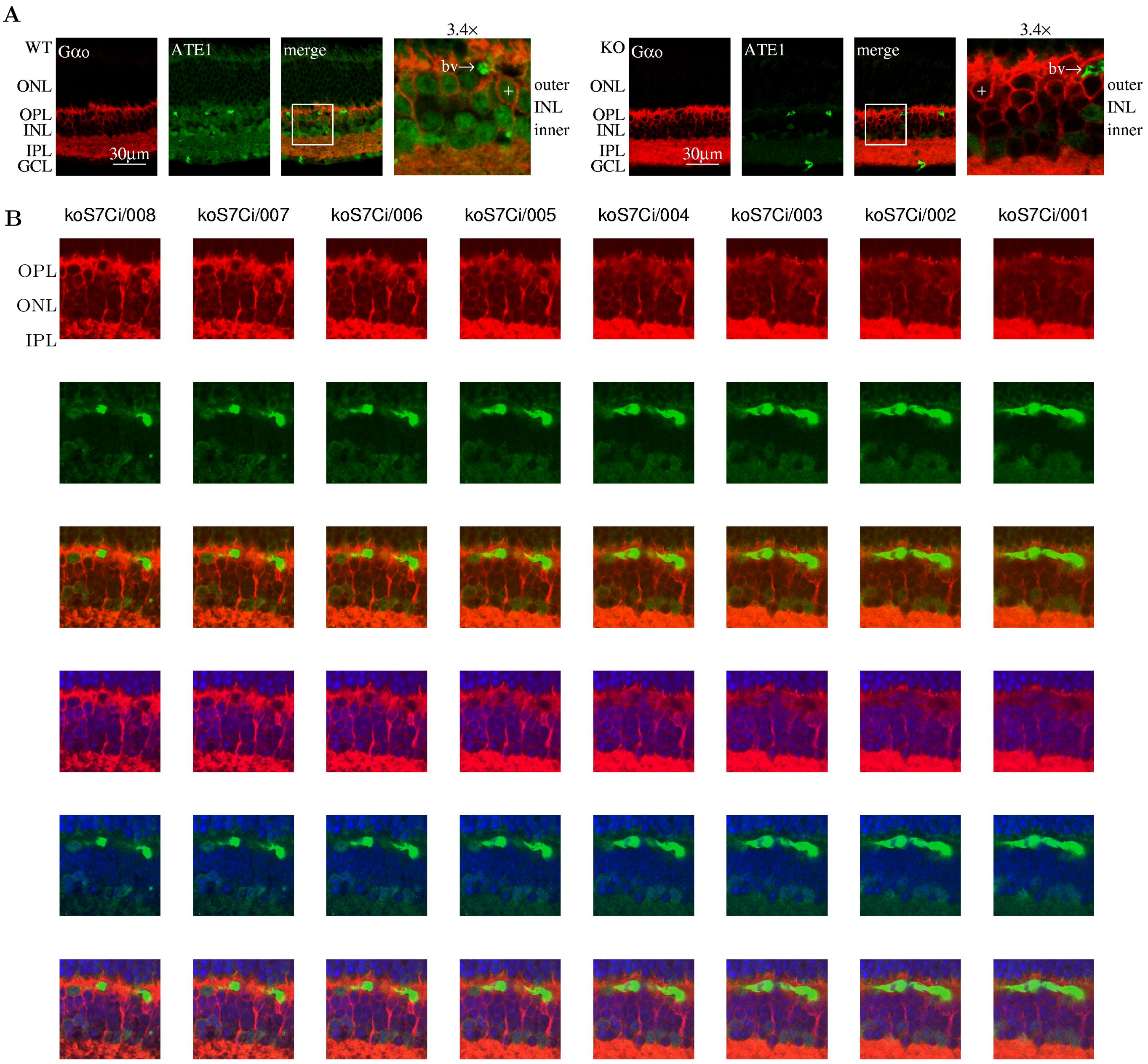

Figure S1: ATE1 expression in the retina. (A) The same retina sections as Figure 1C without DAPI and with Gα_o images subtracted with and scaled by the levels of Gα_o background staining in the ONL of KO and WT, respectively, and ATE1 images subtracted with the level of ATE1 background staining in the ONL of KO. 6 pairs of images of retinal sections like these from WT and KO littermates were used to quantify Gα_o and ATE1 protein levels of marked retina layers shown in Figure 1D and 1E. (B) The serial sections of confocal images of another KO retina ($5\mu m \times 5\mu m$ zoomed window) illustrate that the bright green spots below the OPL are blood vessels and that axons (red lines) extend from ON bipolar cells to IPL. Red: Gα_o, green: ATE1, and blue: DAPI.

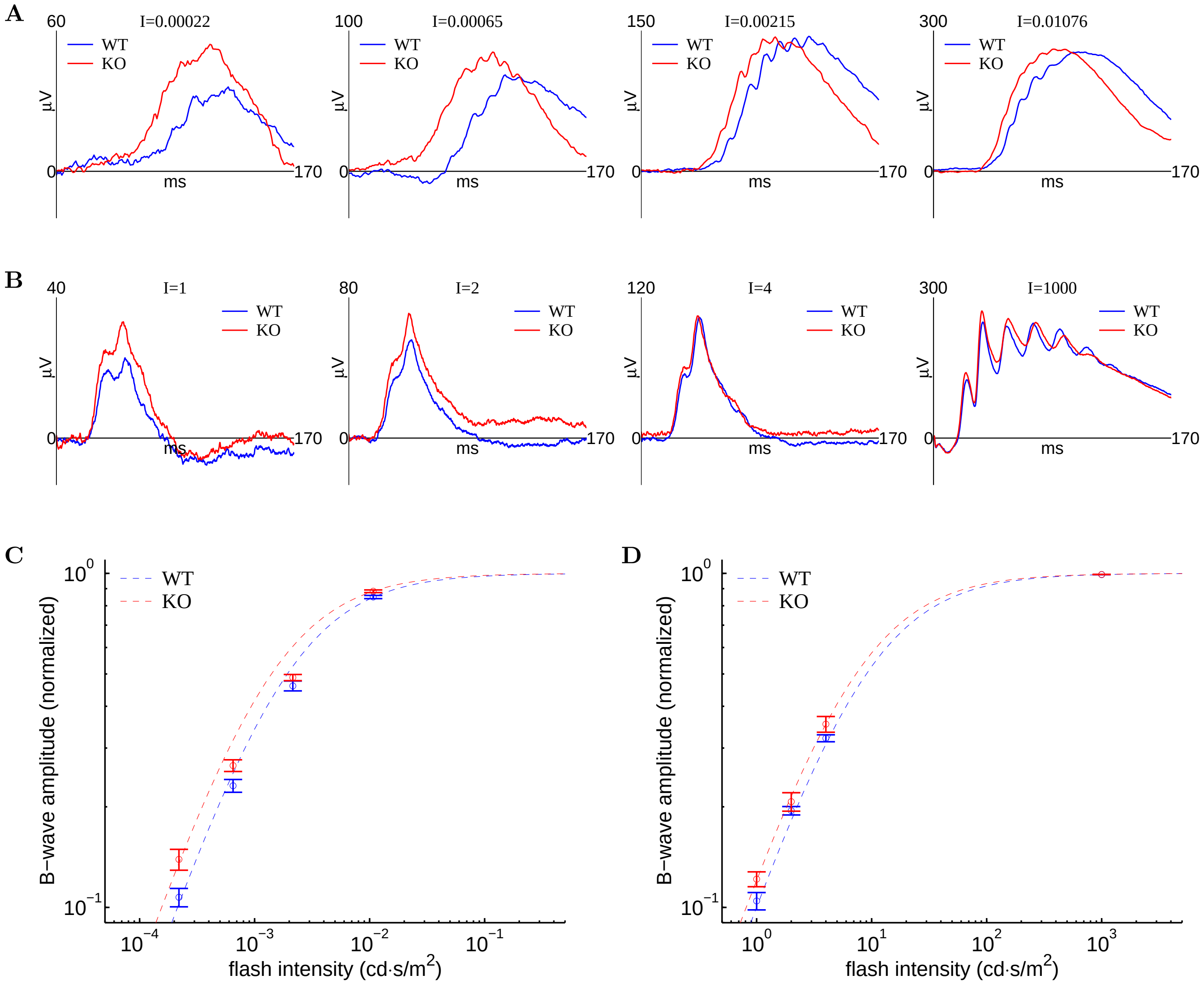

Figure S2: ERG responses of Ate1 KO and WT. (A) A representative series of scotopic B-wave stimulated with the marked flash intensities in $cd{\cdot}s/m^2$. (B) A representative series of photopic B-wave stimulated with the marked flash intensities in $cd{\cdot}s/m^2$. The raw data are plotted here. The first and last plots in (A) and (B) are also presented in Figure 2A-D after 22.5 Hz low-pass filtering. (C, D) The average B-wave amplitude versus the flash intensity. The scotopic (C) and photopic (D) amplitudes were normalized to the best-fit saturating amplitudes of scotopic and photopic B-wave, respectively. The error bars represent $\pm$ SEM (n=7). The curves represent the predictions by the Equation 1 using the corresponding parameters from the means of Figure 2E. Note that we did the photopic experiment with a rod suppressing background.

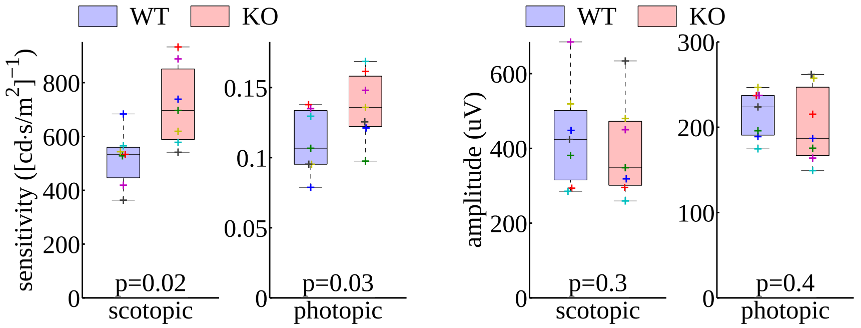

Figure S3: An alternative statistical test to show that lack of Ate1 increases the light sensitivity of ON bipolar cells (left) while the response amplitude does not change (right). Each box shows the lower quartile, median, and upper quartile values. Each pair of data points of the same color and shape represents a WT/KO littermate pair. The p-values are from paired Wilcoxon t-test (Wilcoxon signed-rank test for median). These values are not qualitatively different from the p-values from Student's t-test and do not change the conclusion of Figure 2E and 2F. The maximum length of the whiskers are 1.5 times of the inter quartile range. None of the data points is outside this range.

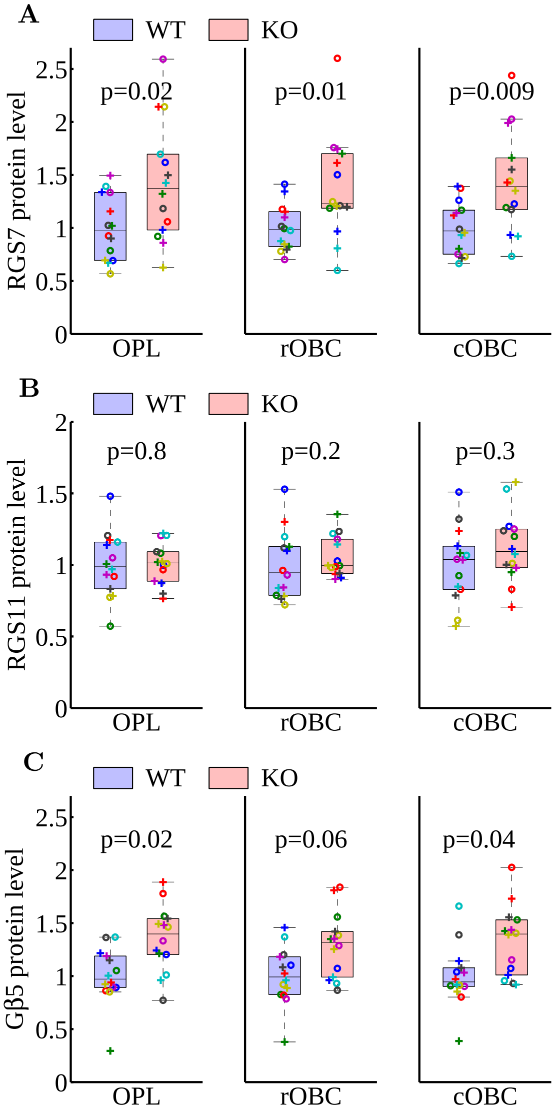

Figure S4: An alternative statistical test to show that RGS7 and Gβ_5 increase in the OPL and in the dendrites of rod-OBCs and cone-OBCs of KO retinas while RGS11 did not change. The p-values here are from paired Wilcoxon t-test (Wilcoxon signed-rank test for median). Each pair of data points of the same color and shape represents a WT/KO littermate pair. Each box shows the lower quartile, median, and upper quartile values. The maximum length of the whiskers are 1.5 times of the inter quartile range. The data points outside this range are treated as outliers. (A) For RGS7 in OPL, rOBC, and cOBC, the p-values including all data points are shown in the plot; and the p-values excluding the outliers are 0.02, 0.03, and 0.02, respectively. (B) There is no outliers for RGS11. (C) For Gβ_5 in OPL, rOBC, and cOBC, the p-values including all data points are shown in the plot; and the p-values excluding the outliers 0.05, 0.06, and 0.005, respectively. These p-values (A), (B), and (C) are not qualitatively different from the p-values from Student's t-test and do not change the conclusion of Figure 5C, F, I, respectively.

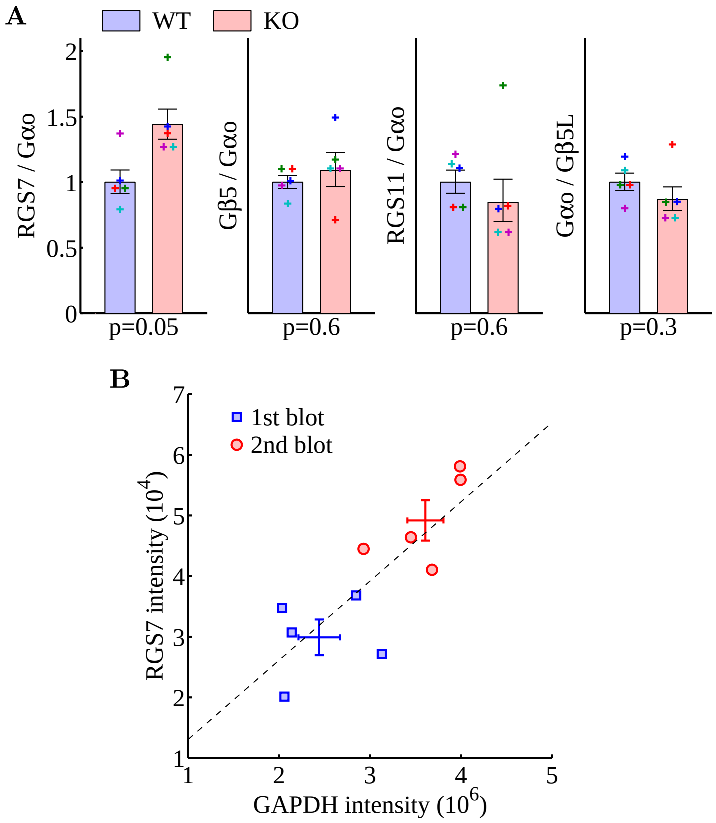

Figure S5: (A) RGS7 protein is enriched in the retina of Ate1 conditional knockout mice. Same as Figure 6D except that the p-values in the plots are from paired Student's t-test for the 5 pairs of littermates from the 3 litters, and the data points from each pair are plotted with a unique symbol. (B) Signal intensities of RGS7 and GAPDH are proportional to loads in the range of interest. The intensity data were from two Western blots with 5 lanes each generated as the following: after 12 hours Rgs7 transfection, one colony of Ate1 KO cells was divided equally into 5, each of which was lysated and then divided into one of the corresponding lanes of the two blots; the lanes within each blot were loaded equally and the 2st blot was loaded with 50% more cell lysate than the 1st blot. The average signal intensities for both RGS7 and GAPDH increased ∼50% for the 2nd blot relative to the 1st blot and the differences between the two blots are significant (paired Student's t-test p=0.002 and p=0.006 for RGS7 and GAPDH, respectively). The error bars represent ± SEM. The dotted line represents the best fit of least-square-error of the 10 data points: $r = 75 \pm 4$ for RGS7=GAPDH/r.

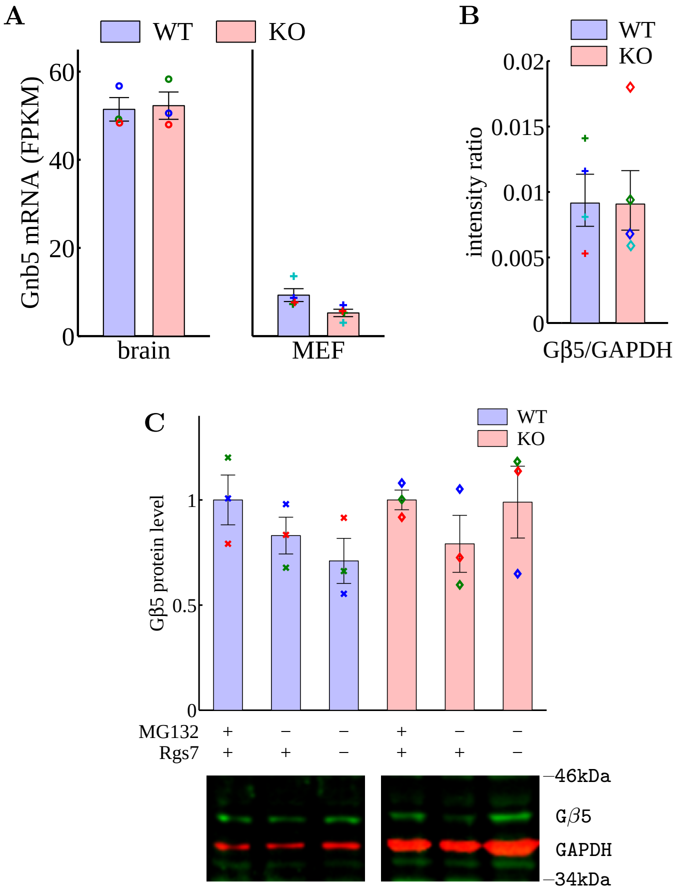

Figure S6: Enodogenous expression of Gβ_5 in mouse embryonic fibroblasts. (A) The Gnb5 mRNA (short) encoding Gβ_5 protein in the mouse brain and the mouse embryonic fibroblast (MEF) from the wildtype (WT) and Ate1 knockout (KO) littermates, expressed in Fragments Per Kilobase of transcript per Million mapped reads (FPKM) from RNA sequencing (see Methods). All levels are significantly greater than zero. The levels are lower in the MEF than in the brain. There is no significant difference between WT and KO. (B) Gβ_5 (relative to GAPDH) endogenously expressed in MEF (corresponding to the condition of the 3rd and 6th lanes of C). They are a fraction of the retinal expression (Figure 6C). There is no significant difference between WT and KO. (C) Western blot quantification of Gβ_5 endogenously expressed in WT and KO MEFs in the Rgs7 tranfection experiments (Figure 7). The effect of MG132 treatment on Gβ_5 protein levels does not depend on the WT or KO background, indicating that its stability is not affected directly by Ate1. Error bars represent SEM. Data points from each pair are plotted with an unique symbol.

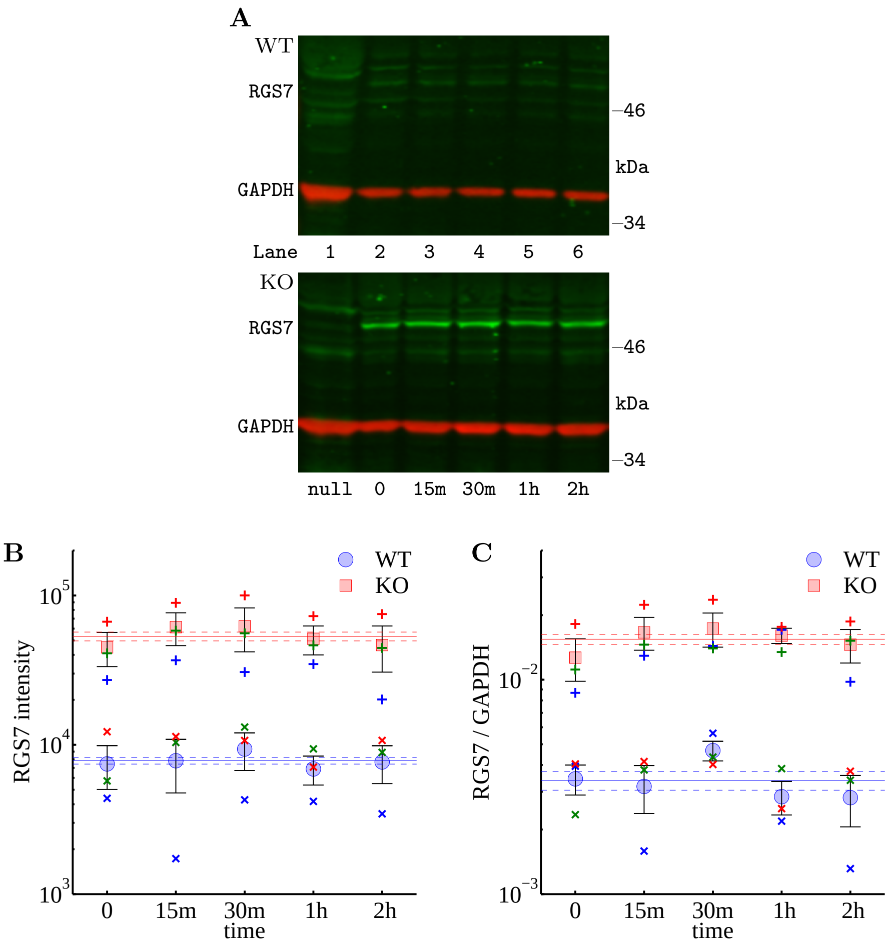

Figure S7: Without MG132, RGS7 protein levels are stable. (A) Western blot of RGS7 exogenously expressed in wild type (WT) and Ate1 knockout (KO) mouse embryonic fibroblasts transfected with Rgs7. 12 hours after transfection (lane 2), cells were further incubated and harvested at the indicated time points. Without MG132, there is no significant difference in protein levels between different time points for both WT and KO cells, in strong contrast to Figure 7A in which RGS7 in WT cells with MG132 changes significantly over time. Lane 1 shows the blots of untransfected control cells. The amounts of RGS7 (B) and RGS7 normalized by GAPDH (C) do not change at different time points. Each data point was the average over 3 technical replicates (individual replicate data are shown in unique color). The error bars represent SEM.

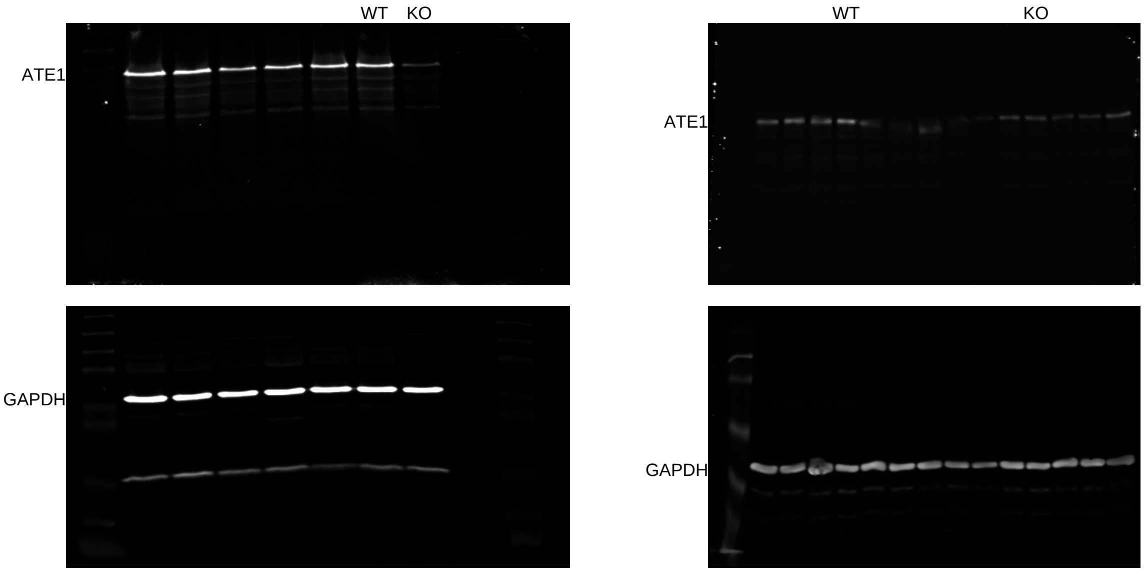

Figure S8: 2 channel images of the full length gels of Western blots for Figure 1B, brain (left) and retina (right).

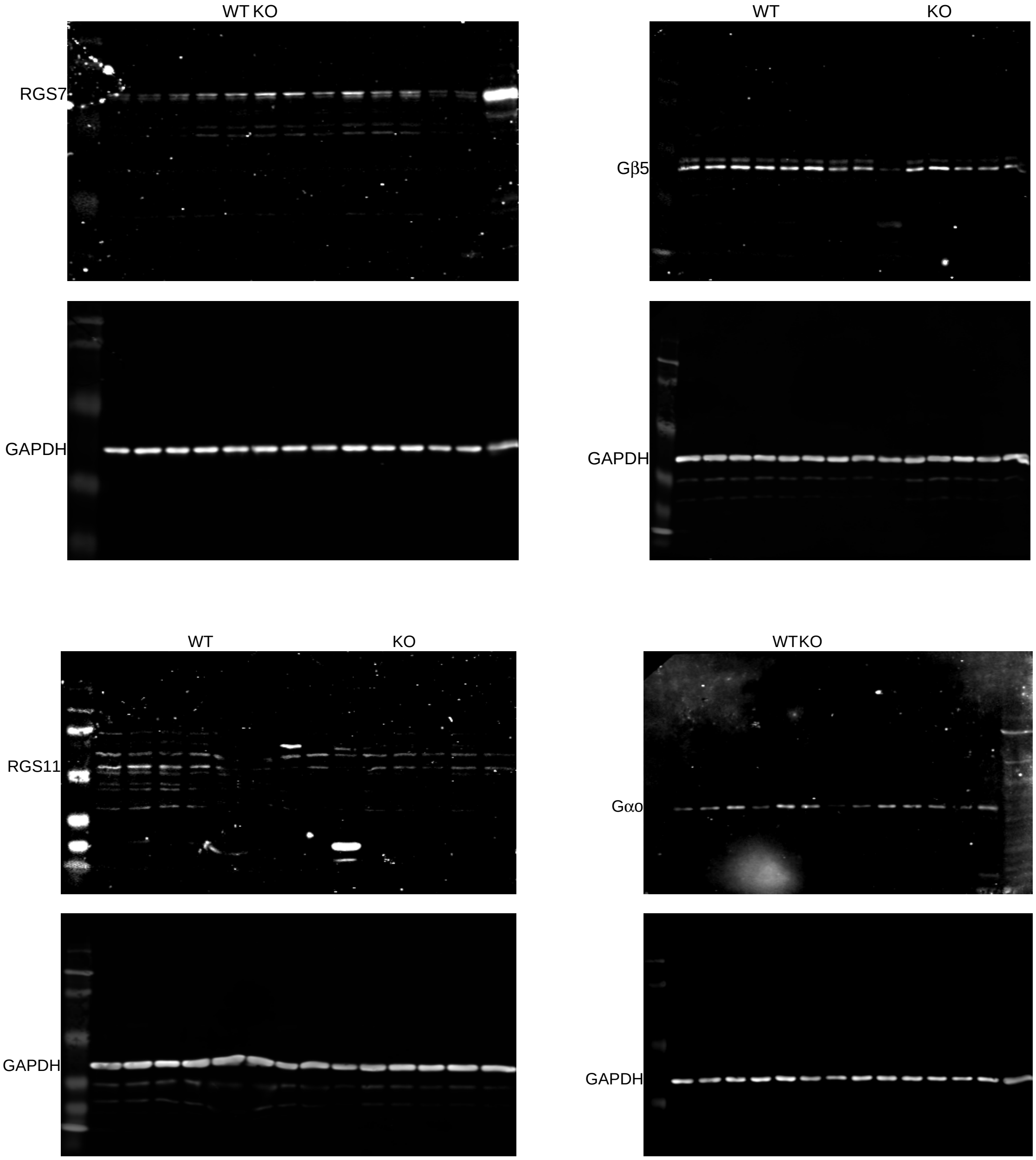

Figure S9: 2 channel images of the full length gels of Western blots for Figure 6C.

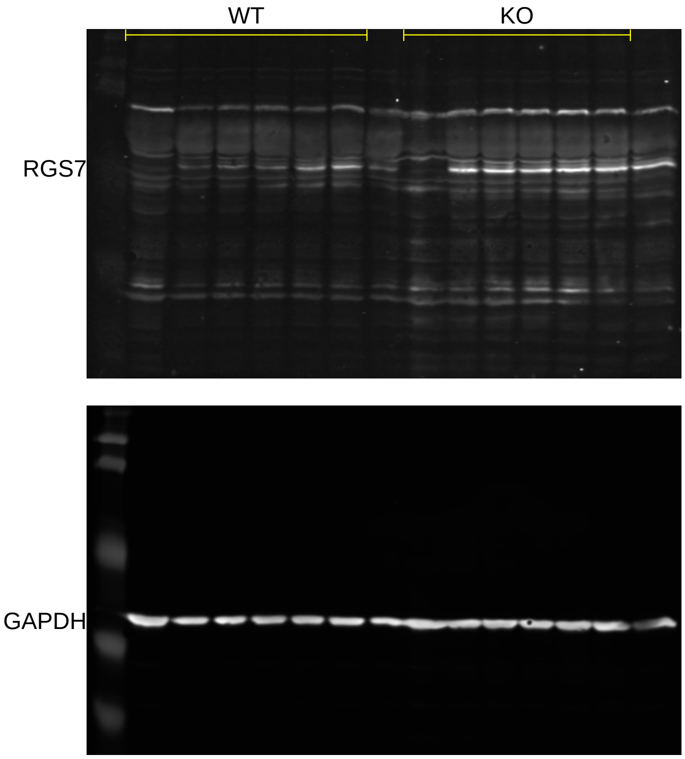

Figure S10: 2 channel images of the full length gel of Western blot for Figure 7A.

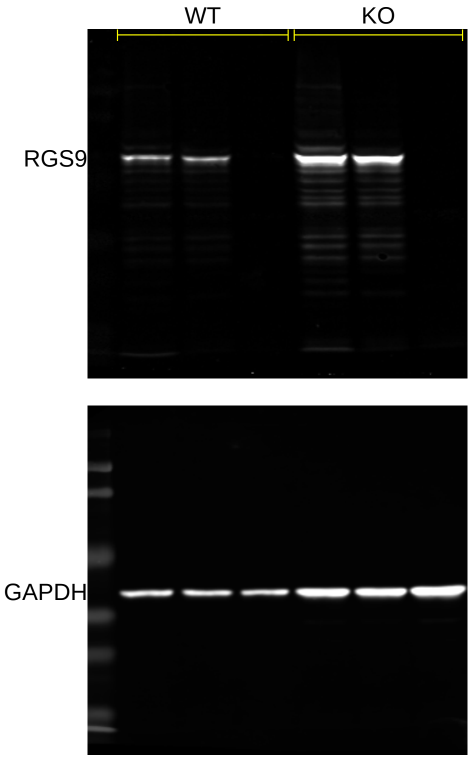

Figure S11: 2 channel images of the full length gel of Western blot for Figure 7C.

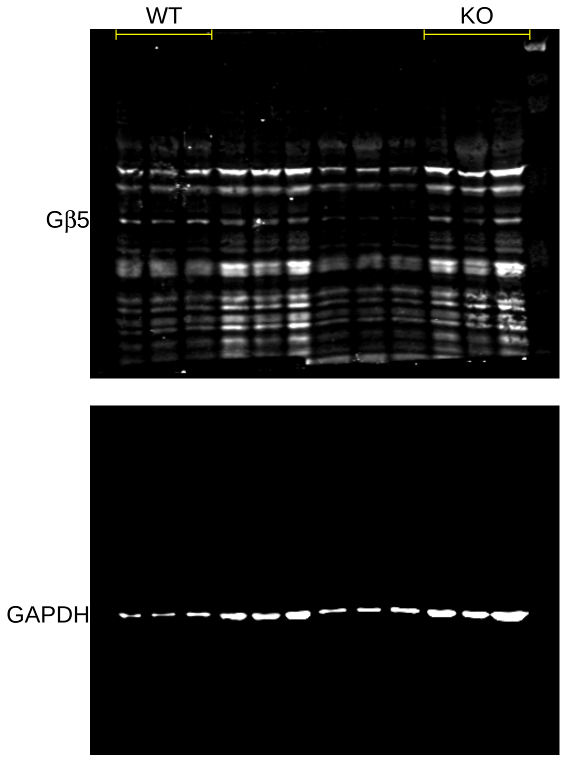

Figure S12: 2 channel images of the full length gel of Western blot for Figure S6C.

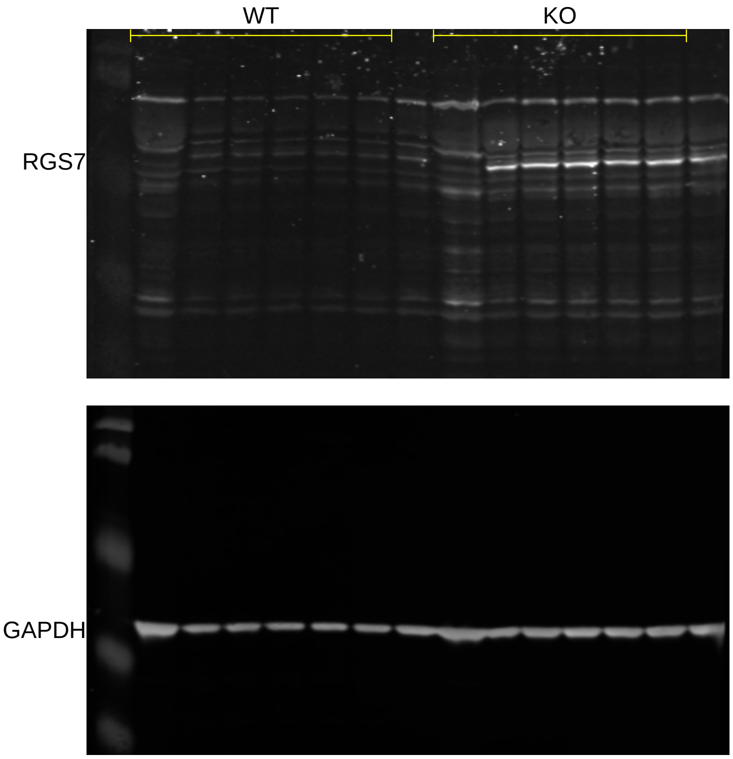

Figure S13: 2 channel images of the full length gel of Western blot for Figure S7A.
